# Cost-effectiveness of preventive COVID-19 interventions: a systematic review and network meta-analysis of comparative economic evaluation studies based on real-world data

**DOI:** 10.7189/jogh.15.04017

**Published:** 2025-02-21

**Authors:** Xiaoyu Tang, Sun Sun, Mevludin Memedi, Ayako Hiyoshi, Scott Montgomery, Yang Cao

**Affiliations:** 1 Clinical Epidemiology and Biostatistics, School of Medical Sciences, Faculty of Medicine and Health, Örebro University, Örebro, Sweden; 2 Department of Epidemiology and Global Health, Umeå University, Umeå, Sweden; 3 Centre for Empirical Research on Information Systems, Örebro University School of Business, Örebro, Sweden; 4 College of Business, Alfaisal University, Riyadh, Saudi Arabia; 5 Clinical Epidemiology Division, Department of Medicine Solna, Karolinska Institute, Stockholm, Sweden; 6 Department of Epidemiology and Public Health, University College London, London, UK; 7 Unit of Integrative Epidemiology, Institute of Environmental Medicine, Karolinska Institute, Stockholm, Sweden

## Abstract

**Background:**

There is a knowledge gap regarding the effectiveness and utility of various preventive interventions during the COVID-19 pandemic. In this study, we aimed to evaluate the cost-effectiveness of various COVID-19 preventive interventions, including non-medical interventions (NMIs) and vaccination programs, using real-world data across different demographic and socioeconomic contexts worldwide.

**Methods:**

We searched Medline, Cochrane Library, Embase, and Web of Science Core Collection from December 2019 to March 2024. We identified 75 studies which compared 34 COVID-19 preventive interventions. We conducted a network meta-analysis to assess the incremental net benefits (INB) of these interventions from both societal and health care system perspectives. We adjusted purchasing power parity (PPP) and standardised willingness to pay (WTP) to enhance the comparability of cost-effectiveness across different economic levels. We performed sensitivity and subgroup analyses to examine the robustness of the results.

**Results:**

Movement restrictions and expanding testing emerged as the most cost-effective strategies from a societal perspective, with WTP-standardised INB values of USD 21 050 and USD 11 144. In contrast, combinations of NMIs with vaccination were less cost-effective, particularly in high-income regions. From a health care system perspective, vaccination plus distancing and test, trace, and isolate strategy were highly cost-effective, while masking requirements were less economically viable. The effectiveness of interventions varied significantly across different economic contexts, underlining the necessity for region-specific strategies.

**Conclusions:**

In this study, we highlight significant variations in the cost-effectiveness of COVID-19 preventive interventions. Tailoring strategies to specific regional economic and infrastructural conditions is crucial. Continuous evaluation and adaptation of these strategies are essential for effective management of ongoing and future public health threats.

**Registration:**

PROSPERO: CRD42023385169.

The global COVID-19 outbreak, triggered by the severe acute respiratory syndrome coronavirus 2 (SARS-CoV-2), has left significant public health challenges and economic turmoil behind. According to reports from the Coronavirus Resource Centre, Johns Hopkins University of Medicine, as of 10 March 2023, there were 676.61 million confirmed diagnoses of COVID-19 and 6.88 million related deaths worldwide [1]. Furthermore, the pandemic has led to substantial economic losses, contributing to increased health care expenses and reduced productivity. The estimated global costs range between USD 77 billion and USD 2.7 trillion [2], while global life expectancy has decreased by 1.6 years – the most significant drop since the Second World War [3,4].

Although the COVID-19 pandemic appears to have faded from the forefront of global headlines, and the World Health Organization (WHO) declared the end of the pandemic phase of COVID-19 [5], the urgency to conduct a cost-effectiveness analysis of interventions remains paramount [6]. As we transition from emergency response to long-term management, understanding the economic efficiency of various interventions is critical. This not only ensures the judicious use of limited resources but also prepares us for future public health emergencies.

Globally, the response to the unprecedented pandemic has included a variety of interventions, reflecting the diverse health care infrastructures, economic capabilities, and demographic profiles across countries [7]. These interventions are broadly categorised into preventive interventions, which include non-medical interventions (NMIs) and vaccination programs, and pharmaceutical interventions or medical treatments, each with unique strategies, cost considerations, and health outcomes [8,9].

The implementation of public health measures, including social distancing [10], testing [11] and tracing [12], lockdowns [13], mask mandates [14], and travel restrictions [15], has been effective in reducing transmission, lowering hospital admission rates, and preventing deaths. These measures have also provided critical time windows for health care systems to prepare, manage caseloads, and support the development and deployment of vaccines [16]. However, these measures also resulted in substantial economic costs stemming from disrupted businesses, decreased workforce participation, and the need for government financial support programs [17].

The development and deployment of medical treatments for COVID-19, including antiviral drugs [18], corticosteroids [19], and monoclonal antibodies [20], have improved survival rates, reduced the severity of symptoms among patients admitted to hospital, and decreased the length of hospital stays. These advancements have required significant investments in research and development, as well as costs related to manufacturing, distribution, and administration.

Global vaccination efforts have significantly curtailed COVID-19 transmission, morbidity, and mortality rates, facilitating the reopening of economies and the easing of public health restrictions [21]. The swift development, manufacture, and dissemination of vaccines have incurred considerable expense, encompassing research and development, mass production, distribution logistics, and the execution of widespread vaccination campaigns [22].

Evaluating the cost-effectiveness of COVID-19 preventive measures is essential for optimal resource allocation. Systematic reviews and meta-analyses are vital for synthesising evidence on the cost-effectiveness of these interventions, providing valuable insights for policymakers and health care providers. Recent systematic reviews have made substantial contributions to understanding the cost-effectiveness landscape of COVID-19 interventions. For instance, the study of Zhou et al., which included 85 modelling studies for systematic review and 25 for meta-analysis, revealed that NMIs, vaccinations, and pharmaceutical interventions were all cost-effective strategies against COVID-19. However, most of the evidence originated from high-income and middle-income countries, underscoring a gap in data from lower-income settings [23]. Elvidge et al. systematic review of 15 studies emphasised the growing number of economic evaluations supporting the prioritisation of interventions such as repurposed antivirals and immunotherapies. This review highlighted the need for head-to-head analyses and considerations of different disease variants, which limits the applicability of conclusions to evolving pandemic contexts.[24] Another recent systematic review by Vardavas et al., which summarised 41 studies focusing on countries within the Organization for Economic Cooperation and Development, indicated that interventions such as testing and screening, vaccination, and social distancing were cost-effective strategies for managing the pandemic. However, the review did not apply meta-analysis to quantify the findings [25].

Therefore, there are knowledge gaps in previous reviews. First, they were limited to specific types of interventions and regions, failing to capture the wide range of strategies employed in various contexts. Second, these reviews did not sufficiently address the variability in health benefit assessments, which can vary significantly across geographical locations and countries. Third, some studies included in previous systematic reviews relied only on simulations and theoretical assumptions rather than incorporating real-world data (RWD), which could lead to discrepancies between predicted and actual outcomes. Furthermore, the dynamic nature of the pandemic, marked by the emergence of new health outcomes and economic data due to the disease’s delayed effects and adaptations in public health practices in response to new virus variants, necessitates the updating of prior analyses.

We aimed to evaluate the comparative cost-effectiveness of COVID-19 preventive interventions, specifically NMIs and vaccination programs, based on their incremental net benefits (INB). INB is a metric used in health economics to determine whether an intervention is cost-effective compared to a standard or alternative measure. It combines both the cost and health outcomes of an intervention into a single value. INB is calculated by subtracting the additional cost of the intervention from the monetary value of the additional health benefit it provides. A positive INB means the intervention offers more benefits than the additional cost, making it cost-effective. Conversely, a negative INB indicates that the costs outweigh the benefits, suggesting the intervention may not be economically viable. INB is useful for policymakers and health care providers as it provides a clear and quantifiable way to assess the economic value of different interventions, helping guide resource allocation decisions [26].

We included studies from a diverse range of demographic and socioeconomic contexts. By incorporating RWD-based health economic studies, leveraging a synthesis of current global evidence, and conducting a network meta-analysis (NMA), our research sought to provide insights into the effectiveness and utility of different preventive interventions at the population level. It may highlight the value and impact of these interventions in managing the pandemic.

## METHODS

We conducted this study in accordance with the recommendations of the Meta-analysis of Economic Studies [27] and we reported the study following the PRISMA-NMA guidelines [28] We registered the study protocol with PROSPERO (CRD42023385169).

### Search strategy

In collaboration with librarians at Karolinska Institute University Library, we developed our search strategy utilising the Population, Intervention, Comparison, and Outcome (PICO) framework [29]. We followed the recommendations from the National Institute for Health and Care Excellence UK [30], using search terms such as ‘COVID-19,’ ‘cost-effectiveness,’ and ‘economic evaluation.’ We refined the search strategy iteratively based on initial results and expert consultation (Appendix S1 in the **Online Supplementary Document**). We included the following databases: Medline, Cochrane Library, Embase, and Web of Science Core Collection. On 4 July 2023, we conducted an initial search for studies published between 1 December 2019 and 30 June 2023, followed by an additional search carried out on 14 March 2024 for studies published between 1 July 2023 and 14 March 2024. We imported all identified references into EndNote, version 20 (Clarivate, Philadelphia, PA, USA), where duplicates were automatically identified and removed before we screened titles and abstracts for relevance.

### Inclusion and exclusion criteria

XT and YC conducted an initial screening of the identified studies based on titles and abstracts to assess their relevance against the predetermined inclusion and exclusion criteria. The inclusion criteria were:

1. Study design: studies published in peer-reviewed journals in English presenting original research, including randomised controlled trials, cohort studies, case-control studies, and observational studies that conduct a cost-effectiveness analysis (CEA), cost-utility analysis (CUA), or cost-benefit analysis (CBA) of preventive COVID-19 interventions;
2. Population: populations of any demographic, including varied age groups, genders, and health status, without geographical restrictions;
3. Intervention and comparison: comparative economic evaluation (CEE) studies that compared two or more preventive interventions against COVID-19, such as vaccination programs and NMIs, including mask-wearing, social distancing, hand hygiene, and lockdown measures;
4. Outcome: studies that reported CEA, CBA, or CUA outcomes, such as incremental cost-effectiveness ratio (ICER), quality-adjusted life years, or other relevant economic evaluation metrics based on real-world cost and/or effectiveness, benefit, or utility data;
5. Timeframe: studies published from December 2019 onwards to ensure relevance to the COVID-19 pandemic context.

At the in-depth screening stage, we excluded articles based on predefined criteria, including relevance to the economic evaluation of preventive COVID-19 interventions, completeness of CEA, and the availability of outcome and real-world data. Specifically, we excluded studies that did not focus on preventive measures, those lacking an economic evaluation component, and those relying solely on theoretical models or simulations instead of real-world evidence. The detailed exclusion criteria were as follows:

1. Non-preventive measures: studies that focused exclusively on the treatment of COVID-19, such as post-infection therapeutic interventions, without analysing preventive measures;
2. Lack of economic evaluation: studies that did not include an economic evaluation component, such as CEA, CUA, or CBA, related to preventive interventions;
3. Incomplete outcome data: studies with incomplete data or results that did not explicitly report the outcomes relevant to the cost-effectiveness of COVID-19 preventive interventions;
4. Theoretical studies: studies that relied on conceptual models and assumptions rather than empirical evidence gathered from real-world settings;
5. Reviews and non-research articles: including systematic, umbrella, scoping, rapid, and narrative reviews, editorials, commentaries, letters, conference abstracts, non-peer-reviewed literature (including grey literature), and other non-research articles;
6. Duplicate studies: multiple reports of the same study or dataset were excluded, with preference given to the most comprehensive or recent report to avoid duplication of data.

### Full-text review and data extraction

Subsequently, we retrieved the full texts of potentially relevant studies from the literature databases for a detailed review to further confirm their suitability for inclusion. We conducted data extraction on CEE studies that met the inclusion criteria. We systematically extracted the relevant data from the eligible CEE studies using a standardised electronic form in Excel, and then we harmonised them by following the recommended steps below [27]. We extracted the following data from the studies:

1. General study characteristics, including year of study published, study period, study setting (country/region), design (model-based, scenario-based, *etc.*), perspective, time horizon, cycle length, discount rate applied to costs and utilities, type of economic evaluation (CEA, CUA, or CBA), economic model used, willingness to pay (WTP) or country-specific cost-effectiveness threshold (if WTP is unavailable);
2. Population characteristics, including sample size and the type of population studied (general population, students, older adults, *etc.*);
3. Preventive COVID-19 interventions compared;
4. Data for estimating INB per person, including monetary cost, effectiveness/utility/benefit measures, *e.g.* life years, quality-adjusted life years, disability-adjusted life years, health-adjusted life years, net monetary benefit, *etc.* incremental costs/effectiveness and/or ICER along with their dispersion measures such as standard deviation (SD), standard error (SE), 95% confidence interval (CI), or variation range, the currency used, and the year of the currency.

Further, we calculated the INB per person using the following formula:



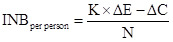



or



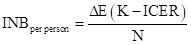



where K is the WTP or one country-specific gross domestic product per capita. ΔE and ΔC represent the differences in effectiveness (*e.g.* quality-adjusted life years, disability-adjusted life years, health-adjusted life years, value of statistical life, and life years) and cost. N refers to the size of the population.

Furthermore, we calculated the variance in the INB for each study. The eligible CEE studies presented their variation parameters for cost, outcomes, and ICER in diverse ways, typically aligning with one of five scenarios as follows:

1. Studies that provided both point estimates and variances for each parameter necessary for calculating the variance of the INB;
2. Studies that reported the means and dispersion measures (SE, SD, 95% CI, *etc.*) for incremental costs, outcomes, and ICER;
3. Studies that offered the means and dispersion measures for costs and outcomes or for ΔC and ΔE but did not report the ICER or its variance;
4. Studies that did not include any dispersion measures but presented a scatter plot of ΔC and ΔE, from which individual data points could be extracted using data extraction software capable of digitising graphs and images [31];
5. Studies that did not provide any dispersion measures or the ΔC – ΔE graph but offered means or point estimates derived from deterministic sensitivity analysis or scenario analysis for costs, outcomes, and ICER.

We estimated the variance in the INB for each scenario differently, as detailed in the recommended practices outlined in the published guidance article [27]. For studies presenting multiple outcomes of a single intervention across various implementation scenarios, we calculated the average INB and pooled variance for the intervention and utilised it in the NMA.

We adjusted all monetary values of the INBs and their variances to 2022 currency values using historical consumer price indices (CPIs) [32–34]. Subsequently, we converted them to USD for the year 2022 using purchasing power parities-adjusted (INB_PPP-adjusted_) USD rates [35], as demonstrated in the following formula [27]:



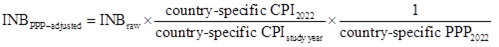



Two reviewers (XT and LH, or YC) independently conducted data extraction for each study. Discrepancies were resolved by discussing and meticulously re-evaluating the pertinent articles.

### Quality assessment

We evaluated the quality of studies using the Joanna Briggs Institute Critical Appraisal Checklist for Economic Evaluations [36]. The 11-item checklist covers key characteristics of health economic analysis, including alternative interventions, costs and outcomes, accuracy and credibility of costs and outcomes, identification of effectiveness, timing of values, incremental analysis, uncertainty and sensitivity analysis, and generalisability. We considered a study that passed nine to 11 items to be of good quality, six to eight items as moderate quality, and fewer than six items as poor quality. Two reviewers (XT and YC) independently conducted the quality assessment process for the study. Any discrepancies were resolved through discussion or consulting a third reviewer (SS).

### Data analysis

We categorised the comparisons extracted for preventive COVID-19 interventions into two health economic perspectives, *i.e.* societal and health care system (including payer and provider). For each perspective, we employed a frequentist NMA approach to integrate the direct and indirect effects of the interventions [37,38].

Unlike multi-arm trials, which provided all pairwise comparisons and may exhibit within-trial correlation, the included CEE studies did not offer all pairwise comparisons. Therefore, we treated the comparisons individually, with their variances estimated independently. This approach ensured a thorough inclusion of all pertinent comparisons.

We constructed network plots and evidence plots to explore the geometry of the intervention network and to depict the direct and indirect comparisons among COVID-19 interventions, illustrating the distribution and density of evidence across different interventions [39].

NMA relies on three key assumptions – transitivity, consistency, and homogeneity – to ensure valid results. Transitivity assumes that studies are comparable across populations, interventions, and outcomes, allowing for indirect comparisons via a common comparator. Consistency is the statistical manifestation of transitivity, which assumes that direct and indirect comparisons yield similar results, with inconsistencies potentially indicating bias. Homogeneity assumes minimal variability among studies comparing the same interventions directly. Transitivity was assessed by comparing study characteristics to ensure that they are similar, such as population demographics, study settings, and outcome measures [40]. We assessed the consistency within the NMA models using the node-splitting method [41]. This approach divides the network estimates into direct and indirect evidence, facilitating the detection and control of inconsistencies in the estimates of individual comparisons [38]. We assessed the heterogeneity using Higgins and Thompson’s *I^2^* statistic, which quantifies the percentage of total variation across studies due to heterogeneity rather than chance. Values of *I^2^*>50% are typically considered indicative of substantial heterogeneity [42]. Subsequently, we employed the random-effects models to synthesise the results from individual comparisons or studies when inconsistency and/or heterogeneity were present [43,44].

We evaluated the risk of bias in the design, conduct, and method of economic evaluations for individual studies using the Joanna Briggs Institute checklist. We used comparison-adjusted funnel plots to evaluate publication bias, and we tested the heterogeneity among studies using the Thompson-Sharp test [45]. Further, we measured the between-study heterogeneity using an adapted DerSimonian-Laird τ^2^, which we then incorporated into the calculation of adjusted random-effects weights [38,46]. These efforts facilitated the identification and mitigation of the impact of such biases on the conclusions of the NMA [47].

The principal summary effect measure in our study was the PPP-adjusted INB per person. We used forest plots to present the results of the comparative cost-effectiveness among interventions, showing the pooled effects and their confidence intervals. Additionally, we ranked interventions using surface under the cumulative ranking curve (SUCRA) values to provide a comprehensive overview of their comparative effects [48,49].

Sensitivity and subgroup analyses were performed to explore the robustness of our findings and investigate potential sources of heterogeneity. In the main sensitivity analysis, we diverged from employing country-specific thresholds and adopted a uniform WTP of USD 50 000 across all countries. This led to calculating an INB standardised by the WTP (INB_WTP-standardised_) [27]. In the second sensitivity analysis, we excluded studies with small sample sizes or those conducted on non-general populations. These approaches allowed us to minimise potential bias resulting from inconsistencies between direct and indirect comparisons, as well as heterogeneity among studies. The subgroup analysis was based on country classifications by income level as defined by the World Bank (Table S1 in the **Online Supplementary Document**) [50]. It enabled us to assess whether the effectiveness of preventive interventions varied across different income levels among economies.

## RESULTS

### Characteristics of the studies

**Figure 1:**
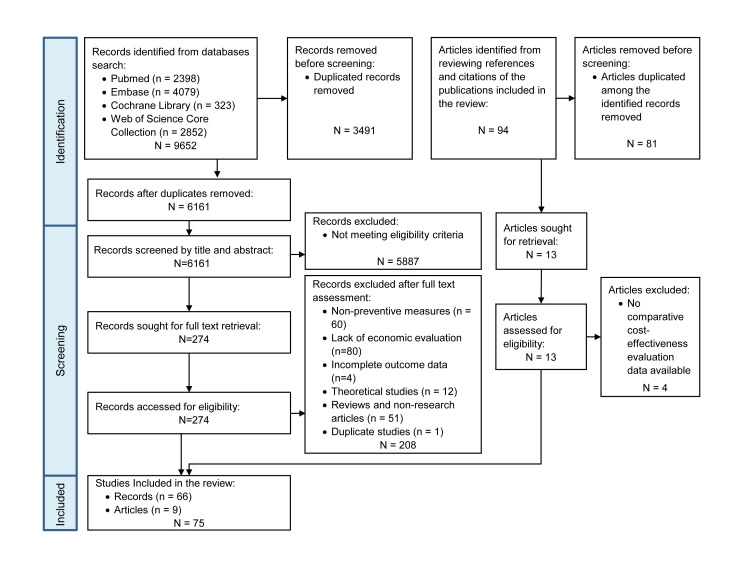
PRISMA flowchart of literature search and screening.

From the four databases searched, we retrieved a total of 9652 articles, of which 3491 were duplicates. Further screening of titles and abstracts led to the exclusion of 5887 articles. Subsequently, we selected 274 articles for full-text review; we excluded 208 due to meeting one or more exclusion criteria. We identified 13 articles by reviewing references and citations of the included studies. Ultimately, 75 studies met the inclusion criteria (**Figure 1**), comparing 41 preventive COVID-19 interventions. After consolidating some interventions due to overlapping definitions, we extracted data for 34 interventions, including a null intervention (NI), and yielding a total of 115 comparisons. All the studies showed good or moderate quality, and we included them in the final analysis. Of the 115 comparisons, 80 were conducted in high-income economies (HIEs), 26 in upper-middle-income economies (UMIEs), nine in lower-middle-income economies (LMIEs), and none in low-income economies (LIEs) (Table S2–3 in the **Online Supplementary Document**).

### Networks of comparisons among preventative COVID-19 interventions

**Figure 2:**
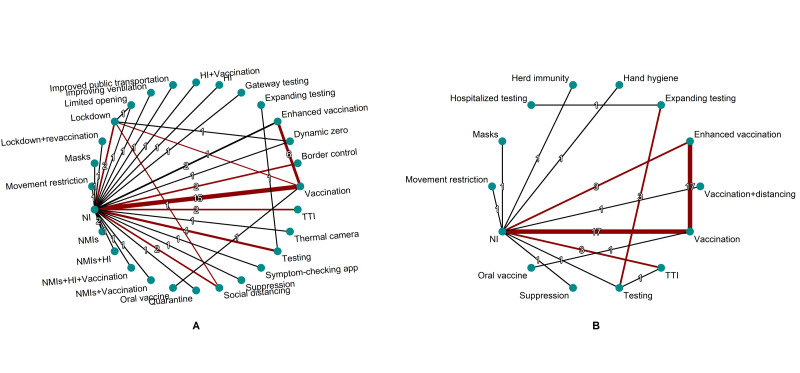
Networks of comparisons among preventive COVID-19 interventions (The constrained network, comprising interventions with two or more comparisons or forming a loop, is highlighted in red). **Panel A.** Societal perspective. **Panel B.** Healthcare system perspective. HI – hospital improvement, NI – null intervention, NMIs – non-medical interventions, TTI – test, trace, and isolate.

We made a total of 61 comparisons among 30 preventive COVID-19 interventions (including NI) from a societal perspective. We excluded two interventions (screening in workplaces *vs.* testing for only severe symptomatic patients) from the NMA because they were not compared with any other 28 interventions (**Figure 2**, Panel A).

From a health care system perspective, a total of 54 comparisons were made among 16 preventive COVID-19 interventions (including NI). The comparison between screening in workplaces and testing for only severe symptomatic patients was again excluded. (**Figure 2**, Panel B).

### Comparative cost-effectiveness of interventions from a societal perspective

**Figure 3:**
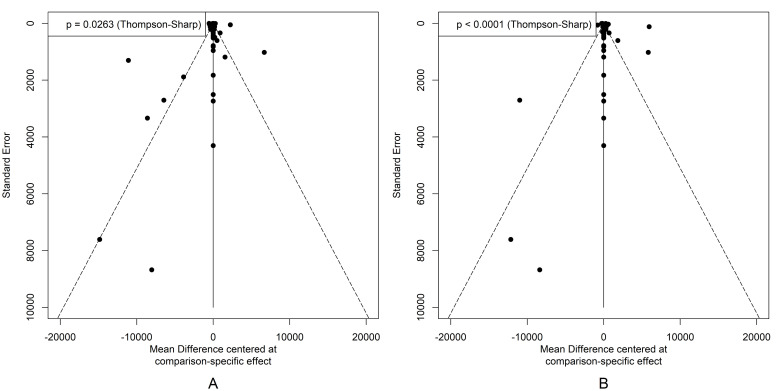
Funnel plots of comparisons among preventive COVID-19 interventions from a societal perspective. **Panel A.** INB_PPP-adjusted_. **Panel B.** INB_WTP-standardised_. INB – incremental net benefit, PPP – purchasing power parity, WTP – willingness to pay.

Funnel plots representing comparisons from a societal perspective indicate potential publication bias, as the asymmetry in the plots suggests variation in effects (**Figure 3**, Panel A–B). This is corroborated by the results of the Thompson-Sharp test (*P* = 0.0263 and *P* < 0.001), which demonstrate a statistically significant deviation from symmetry, suggesting possible publication bias and/or heterogeneity. These observations support the adoption of random-effects models in the analysis.

Due to the extensive number of pairwise comparisons (in total 378 comparisons) among the interventions, we confined our presentation to the comparative cost-effectiveness of other preventive interventions *vs.* NI. Movement restriction had the greatest cost-effectiveness (INB_PPP-adjusted_ = USD 20 976.23), followed by improving ventilation (INB_PPP-adjusted_ = USD 8 581.98) and expanding testing (INB_PPP-adjusted_ = USD 6404.93) (Figure S1, Panel A in the **Online Supplementary Document**). NMIs plus vaccination exhibited the least cost-effectiveness (INB_PPP-adjusted_ = USD –6243.39), with NMIs plus hospital improvement (HI) plus vaccination following closely (INB_PPP-adjusted_ = USD –5342.65). The WTP-standardised results present similar findings, except for the substitution of the third most cost-effective intervention with test, trace, and isolate (TTI) (Figure S1, Panel B in the **Online Supplementary Document**).

Based on the SUCRA ranks, which demonstrate the probability of one intervention being superior across all comparisons within the network, improving ventilation and expanding testing (or TTI) are most likely to be cost-effective. Conversely, NMIs plus vaccination, NMIs plus HI plus vaccination, and NMIs plus HI had the lowest probability of being cost-effective (Figure S2, Panel A–B in the **Online Supplementary Document**).

After excluding two studies with small sample sizes (n = 30, n = 100) [51,52] or those conducted on non-general populations (*e.g.* school students and staff [53,54], the audience of sports competition [55], health care workers [56]), the sensitivity analysis for INB_WTP-standardised_ data suggested that movement restriction, expanding testing, and testing or TTI could be the most cost-effective interventions (Figure S3, Panel A–B in the **Online Supplementary Document**).

Since many interventions were solely compared once, leading to most pooled effects being indirect, we further investigated interventions involving two or more comparisons (**Figure 2**, Panel A). Despite only one direct comparison between lockdown and vaccination or social distancing, we included the two comparisons due to their formation of two loops involving NI and vaccination or social distancing. Figure S4 in the **Online Supplementary Document** illustrates the collective and separate effects of the interventions *vs.* NI along the highlighted comparisons. Social distancing (INB_PPP-adjusted_ = USD 2008.31) and enhanced vaccination (INB_PPP-adjusted_ = USD 571.10) exhibited the highest cost-effectiveness, while lockdown (INB_PPP-adjusted_ = USD –236.69) and testing (INB_PPP-adjusted_ = USD –95.89) were the least cost-effective (Figure S4, Panel A in the **Online Supplementary Document**). The WTP-standardised results align similarly, except for the most cost-effective intervention being TTI (INB_WTP-standardised_ = USD 4821.45) (Figure S4, Panel B in the **Online Supplementary Document**). The total and separate effects of the interventions within the highlighted network compared to each other from a societal perspective are presented in Figure S5 in the **Online Supplementary Document**. Additionally, as illustrated in the majority of the effects are indirect (Figure S6 in the **Online Supplementary Document**).

### Comparative cost-effectiveness of interventions from a health care system perspective

Funnel plots illustrating comparisons from a health care system perspective (Figure S7, Panel A–B in the **Online Supplementary Document**) indicate heterogeneity among the studies, particularly evident in the significantly varied effects for studies with relatively smaller uncertainties. Although the Thompson-Sharp test did not yield statistical significance for the INB_WTP-standardised_ (*P* = 0.9492), the observed dispersion in effect sizes underscores the presence of heterogeneity across the included studies.

Suppression demonstrated the highest cost-effectiveness (INB_PPP-adjusted_ = USD 2729.98), followed by movement restriction (INB_PPP-adjusted_ = USD 2669.27) (Figure S8, Panel A in the **Online Supplementary Document**). Mask wearing exhibited the least cost-effectiveness (INB_PPP-adjusted_ = USD –3391.39), followed by hospitalised testing (INB_PPP-adjusted_ = USD –261.96). The WTP-standardised results revealed distinct findings, where vaccination plus distancing, TTI, expanding testing, hospitalised testing, and testing emerged as highly cost-effective interventions (INB_WTP-standardised_>USD 10 000) (Figure S8, Panel B in the **Online Supplementary Document**).

The SUCRA ranks indicated that, from a health care system perspective, suppression and herd immunity are the most likely to be cost-effective, while hand hygiene, testing, and mask-wearing are the least (Figure S9, Panel A in the **Online Supplementary Document**). However, the WTP-standardised results revealed that vaccination plus distancing and TTI exhibited the highest superiority, followed by expanding testing, testing, and hospitalised testing (Figure S9, Panel B in the **Online Supplementary Document**).

The sensitivity analysis following study exclusion revealed significant changes in the results. TTI, vaccination plus distancing, expanding testing, hospitalised testing, and testing emerged as nearly equally cost-effective interventions, with INB_WTP-standardised_ around USD 30 000 (Figure S10, Panel A–B in the **Online Supplementary Document**). When conducting a constrained network analysis within the interventions involving two or more comparisons, enhanced vaccination (INB_PPP-adjusted_ = USD 257.69) and vaccination (INB_PPP-adjusted_ = USD 154.60) demonstrated the greatest cost-effectiveness compared to NI, while testing exhibited the least (INB_PPP-adjusted_ = USD –126.61) (Figure S11, Panel A in the **Online Supplementary Document**). However, the WTP-standardised results indicated that TTI exhibited the highest cost-effectiveness, with an INB_WTP-standardised_ value of USD 25 560.52 (Figure S11, Panel B in the **Online Supplementary Document**). The total and separate effects of the interventions within the constrained network, compared to each other from a health care system perspective, are illustrated in Figure S12 in the **Online Supplementary Document**. The majority of the effects are indirect (Figure S13 in the **Online Supplementary Document**).

### Subgroup analysis

From a societal perspective, improving ventilation, expanding testing, and dynamic zero had the greatest cost-effectiveness for HIEs, with both PPP-adjusted and WTP-standardised INBs ranging between USD 3000–8000. Conversely, NMIs or their combinations demonstrated the lowest cost-effectiveness, with INBs ranging between USD –6000 to –5000 (Figure S14, Panel A–B in the **Online Supplementary Document**). In UMIEs, movement restriction and TTI demonstrated the highest cost-effectiveness, with INBs potentially reaching as high as USD 67 369.37 (WTP-standardised), while vaccination and border control exhibited the lowest cost-effectiveness, with INBs potentially as low as USD 8.91 (PPP-adjusted) (Figure S14, Panel C–D in the **Online Supplementary Document**). In LMIEs, only vaccination was compared with NI, yielding an INB_PPP-adjusted_ value of USD 117.03 and an INB_WTP-standardised_ value of USD 411.71 (Figure S14, Panel E–F in the **Online Supplementary Document**).

From a health care system perspective, among HIEs, suppression and vaccination plus distancing demonstrated the greatest cost-effectiveness, with PPP-adjusted INBs exceeding USD 2000 (Figure S15, Panel A in the **Online Supplementary Document**). Additionally, vaccination plus distancing could have a WTP-standardised INB as high as USD 31556.73 (Figure S15, Panel B in the **Online Supplementary Document**). In UMIEs, TTI and testing demonstrated greater cost-effectiveness than movement restriction alone and vaccination, potentially exceeding 10-fold in both PPP-adjusted and WTP-standardised INBs (Figure S15, Panel C–D in the **Online Supplementary Document**). For LMIEs, only four interventions were compared with NI. Interventions involving vaccination exhibited greater cost-effectiveness than hand hygiene and mask-wearing, with the latter showing negative INBs (Figure S15, Panel E–F in the **Online Supplementary Document**).

## DISCUSSION

The COVID-19 pandemic has posed unprecedented challenges to public health, economics, and social structures globally. Preventive strategies offer a high benefit-cost ratio by preventing disease transmission and reducing the need for costly treatments. Additionally, they support economic stability by mitigating the need for disruptive lockdowns [22,57,58]. However, prior systematic reviews have often focused solely on specific interventions, such as vaccines [58] or masks [59] or lacked quantified or standardised measurements of cost-effectiveness [25], limiting their utility in policy decision-making.

To the best of our knowledge, this is the first NMA that comprehensively evaluated the cost-effectiveness of preventive COVID-19 interventions. We encompassed interventions ranging from masks, highly recommended at the beginning of the pandemic, to vaccination boosters during the latter stages. This analysis revealed significant variations in interventions and their health and economic impacts across different settings.

From a societal perspective, movement restrictions and expanding testing demonstrated the highest cost-effectiveness, with INB_WTP-standardised_ values of USD 21 050.49 and USD 11 144.11, respectively. In contrast, NMIs and its combinations with vaccination or HI showed the least cost-effectiveness (INB_WTP-standardised_ around USD –6000), a finding observed in the sensitivity analysis (Figure S3, Panel A in the **Online Supplementary Document**). These results suggest potential inefficiencies or mismatches in resource allocation; as of November 2021, less than a quarter of vaccine doses were administered in LMIEs and LIEs despite they bearing a disproportionate burden of morbidity and mortality from the pandemic [60]. The inequitable distribution and access to vaccines might cause excess deaths and further economic, social, and political disruption.

From a health care system perspective, TTI emerged as the most cost-effective intervention (INB_WTP-standardised_ = USD 31 764.36), followed by vaccination plus distancing (INB_WTP-standardised_ = USD 31 556.73 (Figure S10, Panel A in the **Online Supplementary Document**)). Conversely, masks were the least cost-effective (INB_WTP-standardised_ = USD –3330.75 (Figure S8, Panel A in the **Online Supplementary Document**), corroborated by the PPP-adjusted USD – 3391.39 (Figure S10, Panel A in the **Online Supplementary Documen**t) in the sensitivity analysis. This raises questions about their economic viability when broadly implemented without targeting specific settings or populations. The effectiveness of masks can vary widely based on the type used (*e.g.* cloth *vs.* surgical *vs.* N95/filtering facepiece 2) [61]. Higher-quality masks generally offer better protection but are also more expensive and less available. When assessing the increased benefit of high-priced masks, they may not offer favourable cost-effectiveness compared to other measures such as vaccinations and strict suppression [62,63].

In general, movement restrictions, TTI, vaccination plus distancing, and expanding testing emerged as the most cost-effective interventions. Their effectiveness stems from significantly reducing transmission rates, preventing outbreaks from escalating, and alleviating the health care system’s burden. They directly curb new infections and hospital admissions, proving highly effective in periods or areas without vaccine availability. Additionally, they can be rapidly implemented and adjusted based on the pandemic’s severity and dynamics, providing adaptable responses to changing conditions [8]. Although our results are not directly comparable to those of previous meta-analyses due to differences in grouping methods, the INBs for suppression are quite similar, both approximately USD 2000 [23].

Our findings also revealed that the cost-effectiveness of COVID-19 preventive interventions varies widely across different economic levels. From a societal perspective, improving ventilation, expanding testing, and dynamic zero appear to be the most cost-effective in HIEs, while TTI and movement restrictions are more cost-effective in UMIEs (Figure S14 in the **Online Supplementary Document**). From a health care system perspective, vaccinations are most cost-effective in HIEs and LMIEs, while TTI and testing strategies prove most cost-effective in UMIEs (Figure S15 in the **Online Supplementary Document**). The differences identified may be attributed to the following factors.

HIEs typically have more financial resources and advanced health care infrastructure, enabling them to invest in and sustain more resource-intensive interventions like improving ventilation and expanding testing. The measures often require significant upfront investments and ongoing maintenance [51,64,65], which are more feasible in HIEs. While UMIEs may not have the same level of resources as HIEs, they often have sufficient infrastructure to implement moderately resource-intensive strategies effectively. TTI and testing strike a balance between resource use and effective control of virus spread, without the higher costs associated with more resource-intensive interventions [66,67]. LMIEs benefit most from vaccinations because they provide a high return on investment in terms of disease prevention relative to their cost. Vaccinations require less continuous resource expenditure compared to interventions like dynamic zero or expanding testing programs, making them sustainable options for LMIEs with more limited health care funding [68,69].

These results highlight those interventions such as movement restrictions and TTI are highly cost-effective during the pandemic. Health policies should prioritize these interventions in initial outbreak stages and in settings where vaccine availability is low. These measures are particularly effective in preventing the escalation of outbreaks and can be quickly implemented and adjusted based on real-time data about the pandemic's spread and severity [70]. However, the adoption and success of these interventions can vary widely due to differences in public acceptance, governmental support, and logistical capabilities. For example, while movement restrictions may be highly effective in one region, they may not be as successful in another due to variations in enforcement and public compliance [71]. Equitable access to interventions, particularly vaccines, is crucial, especially in lower-income settings where distribution challenges can exacerbate disparities in health outcomes. Policies should also remain flexible to adapt to pandemic dynamics, invest in health care infrastructure, and promote public health education to enhance compliance and effectiveness across diverse global contexts [16,72].

Our study provides a comprehensive evaluation of the cost-effectiveness of COVID-19 preventive interventions using real-world data, setting it apart from previous systematic reviews that often relied on pairwise comparisons or narrative summaries. This analysis not only incorporates a broad range of global data but also employs network meta-analysis to compare interventions across diverse contexts, adding depth to the existing body of research. For instance, a previous meta-analysis by Zhou et al. focused primarily on modelling studies and specific types of interventions [23]. While that study identified NMIs and vaccinations as broadly cost-effective, our findings emphasize that the combination of NMIs and vaccination programs is less cost-effective from a societal perspective in high-income regions (Figures S1, S3, and S14 in the **Online Supplementary Document**) – an essential insight for global policymakers aiming to optimize resource allocation.

Two other reviews, by Elvidge et al. [24] and Vardavas et al. [25], summarised the cost-effectiveness of interventions or treatments and suggested that rapid antigen tests, personal protective equipment, and vaccination strategies were cost-effective. However, these studies did not provide quantified or synthesised results. In contrast, our network meta-analysis not only quantified the comparative effectiveness of interventions but also ranked them by INB, offering a clearer framework for policy recommendations. This analysis sheds new light on the relative value of movement restrictions and expanding testing, which emerged as the most cost-effective interventions from a societal perspective, while combinations of NMIs and vaccination were less efficient in certain contexts.

Additionally, our analysis, with adjustment for WTP and PPP, allows for a more nuanced and globally relevant understanding of cost-effectiveness, which was lacking in previous reviews when comparing across countries.

In summary, our study addresses critical gaps in the literature by offering a real-world, comparative economic evaluation of COVID-19 preventive interventions across diverse socioeconomic settings. By applying network meta-analysis, we extend the scope of previous reviews, providing both region-specific insights and a global perspective on resource allocation strategies during the pandemic.

### Strengths and limitations

Our study has several strengths. We focused on NMIs and vaccination programs, comparing their cost-effectiveness using INB per person across a broad range of contexts and demographics from both societal and health care system perspectives [73]. We meticulously detailed specific interventions and extracted all head-to-head (or direct) comparisons as thoroughly as possible, which can be considered to represent the ‘pure’ cost-effectiveness of each specific intervention.

To enhance the comparability of the monetary values of interventions, we employed two methods to harmonise the INB. First, we converted different INBs into USD using PPP instead of traditional exchange rates, facilitating more accurate country comparisons. Second, in our sensitivity analysis, we further standardised the INB across countries using a standardised WTP of USD 50 000 to make life values more comparable. While some results are sensitive to the WTP setting and utility estimation, the findings from a societal perspective for most interventions are notably robust (Figure S1 in the **Online Supplementary Document**).

By leveraging the capabilities of NMA, we were able to simultaneously compare multiple interventions within a single analysis. This approach, integrating both direct and indirect comparisons across a network of studies, significantly enhanced our statistical power to discern differences among interventions [40]. We also provided a ranking of these interventions based on their effectiveness, filling gaps identified in earlier reviews and offering critical insights for policymakers and health care providers to guide the effective allocation of resources in the fight against future pandemics.

Additionally, our subgroup analysis acknowledges the profound influence of socioeconomic contexts on the effectiveness and cost-effectiveness of interventions. Strategies that are feasible and effective in high-income countries may not be suitable for lower-income settings. This socioeconomic heterogeneity must be considered when planning and implementing public health strategies, as it affects both the feasibility and the impact of interventions.

Our study has several limitations. First, the ambiguity in defining interventions and the complexity of their adoption were not fully addressed. For example, some interventions, such as suppression, NMIs, TTI, and dynamic zero, are combinations of several interventions rather than standalone measures. Additionally, for interventions like testing, differentiating between testing methods (polymerase chain reaction or antigen) and the frequency and timing of testing was difficult or even impossible. These factors complicate the comparison of interventions and the interpretation of results.

The dynamic nature of the COVID-19 pandemic, characterised by varying transmission rates, evolving viral strains, and fluctuating public health policies, also complicates the assessment of intervention effectiveness. Alongside, the virus has resulted in long-term morbidity among survivors, particularly those experiencing ‘long COVID,’ which encompasses a spectrum of enduring symptoms, including mental health issues such as anxiety, depression, and stress [74]. These conditions are often aggravated by factors such as isolation and economic instability [75]. These complexities necessitate continual or longitudinal cost-effectiveness assessments, which go beyond the methodology of a conventional meta-analysis.

Another limitation is that we were unable to account for the impact of different viral variants, such as Delta or Omicron, on the cost-effectiveness of the preventive interventions. Most of the included studies did not specify which variant was predominant during their analysis, which limited our ability to differentiate the effectiveness under variant-specific conditions. Since the effectiveness of interventions is likely to vary depending on the viral strain, future studies with detailed variant information would allow for a more precise evaluation of how cost-effectiveness may shift in response to emerging variants.

Although we endeavoured to make monetary and life values comparable, the diversity in pricing, cost compositions, and estimation methods in CEA, CBA, and CUA studies were not standardised, leading to significant uncertainty in cost-effectiveness evaluations that were not fully captured in our study. Estimating the costs and utilities associated with each intervention involves assumptions that may not hold true across different contexts or over time. Costs can vary due to regional differences in health care pricing, labour costs, and the availability of technology. Similarly, the perceived utility of an intervention may vary among different populations, affecting the generalisability of the results.

The estimation of uncertainty in cost-effective measures is diverse, with various methods, such as confidence intervals, sensitivity analyses, or probabilistic modelling. This diversity was not captured in the overall pooling of effects.

Unfortunately, as with previous meta-analyses, we did not identify cost-effectiveness analyses from LIEs. This gap highlights the urgent need for targeted research to better understand how interventions perform in these under-researched regions. Future studies should focus on cost-effectiveness analyses in LIEs, supported by international collaboration and improved data collection efforts. Additionally, building infrastructure for routine health care monitoring systems in LIEs would enhance the availability of real-world evidence, enabling a more comprehensive understanding of cost-effectiveness in these specific contexts. These efforts are crucial for developing effective and equitable health policies that address the unique economic and infrastructural challenges faced by LIEs.

Furthermore, while this study leverages RWD to improve the applicability of its findings, there are inherent limitations associated with RWD that must be acknowledged. Data quality can vary widely between countries and health care systems, leading to inconsistencies in reporting and potential biases. Incomplete records, differences in data collection methods, and varying reporting standards can all have an impact on the reliability and comparability of the results. These challenges are particularly pronounced when integrating data from diverse economic contexts, where the availability and accuracy of health care data may vary significantly. Recognising these limitations underscores the importance of improving data collection practices, standardising reporting, and expanding RWD infrastructure, particularly in lower-income settings, to ensure more robust and comprehensive analyses in future research.

These limitations highlight the need for flexibility and context-specific approaches in the cost-effectiveness evaluation of pandemic response strategies. To improve the robustness of cost-effectiveness analyses in such contexts, future research should aim to standardise intervention definitions and enhance the accuracy of cost and utility estimates. Furthermore, conducting more direct comparisons and designing studies tailored to specific socioeconomic contexts would deepen the data pool, enabling more precise and contextually relevant policy recommendations.

## CONCLUSIONS

Our NMA underscores the profound and varied impacts of COVID-19 interventions worldwide. While these strategies have imposed a substantial economic burden, their benefits in saving lives and reducing further economic impacts highlight their critical importance. From a societal perspective, movement restrictions and expanding testing have emerged as the most cost-effective strategies, significantly easing health care burdens. However, combining NMIs with vaccinations has been shown to be less cost-effective, indicating potential mismatches in resource allocation – issues that are particularly pressing in regions with delayed vaccine distribution. From a health care system perspective, TTI and vaccination plus distancing have been identified as highly cost-effective, whereas the broad, non-targeted implementation of masks has proven less economically viable. However, due to only two studies identified that evaluated the cost-effectiveness of the masks (one for surgical and N95 masks [14] and one for standardised high-filtration masks [53]), we grouped them together as a single category and did not differentiate between mask types. To avoid oversimplification, an in-depth analysis with additional data are needed in the future to provide policymakers with more practical insights into the variations in costs and effectiveness of different mask types, with further details of how they were used.

Future research should focus on refining the definitions of interventions, improving cost and utility estimates, and conducting studies in low-income economies to enhance the global applicability and equity of pandemic response strategies. Continuous evaluation and adaptation of intervention strategies, informed by cost-effectiveness analysis, are essential as the global community continues to navigate the ongoing challenges of the pandemic and prepare for future public health threats.

When considering future pandemics, the relevance of these findings may vary based on factors such as the type of organism, incubation time, mode of transmission, transmissibility, and fatality rate. It is crucial to note that these strategies are primarily relevant to diseases transmitted through droplet-based airborne transmission. For pathogens with different transmission modes, such as contact or vector-borne diseases, the cost-effectiveness of interventions may differ significantly. Therefore, the effectiveness and economic viability of interventions should be tailored to the specific characteristics of each pathogen and the unique economic and infrastructural conditions of each region.

## Additional material


Online Supplementary Document
