# Supplementary material for: Cost-effectiveness of preventive COVID-19 interventions: a systematic review and network meta-analysis of comparative economic evaluation studies based on real-world data
Source: J Glob Health. 2025 Feb 21;15:04017. doi: 10.7189/jogh.15.04017 (PMC11842005; doi:10.7189/jogh.15.04017)
Supplement: Online Supplementary Document [file jogh-15-04017-s001.pdf]

Supplement to: Tang X, Sun S, Memedi M, Hiyoshi A, Montgomery S, Cao Y. Cost-effectiveness ofpreventive COVID-19 interventions: a systematic review and network meta-analysis of comparative economicevaluation studies based on real-world data. J Glob Health. 2025;15:04017.

Documentation of search strategies
University Library search consultation group

Date: March 14, 2024

Topic/research question: Costs (including direct medical costs and productivity loss) and effectiveness associated with different anti-pandemic strategies/interventions?

Name of researcher(s): Yang Cao, Institute of Environmental Medicine

Librarian(s): Emma-Lotta Säätelä and Ingrid Andersson

Databases:

1. Medline (Ovid)
2. Cochrane Library (Wiley)
3. Embase (embase.com)
4. Web of Science Core Collection (Clarivate)

Total number of hits:

- Before deduplication: 9 652 (8 127 July 2023)
- After deduplication: 6 161 (5178 [July 2023] + 983 [March 2024])

Search strategy

A literature search was performed in the following databases: Medline (Ovid), Cochrane Library (Wiley), Embase (embase.com) and Web of Science Core Collection (Clarivate). The last search was conducted 2024-03-14.

After the original search was performed on 4 July 2023, the search was last updated on 14 March 2024 using the methods described by Bramer et al. (1)

The search strategy was developed in Medline (Ovid) in collaboration with librarians at the Karolinska Institutet University Library. For each search concept Medical Subject Headings (MeSH-terms) and free text terms were identified. The search was then translated, in part using Polyglot Search Translator (2), into the other databases.

Language restriction was made to English.

Articles from before the pandemic (e.g. older than 2019) were not reviewed.

De-duplication was done using the method described by Bramer et al (3). One final, extra step was added to compare DOIs.

References

1. Bramer W, Bain P. (2017). Updating search strategies for systematic reviews using EndNote. *Journal of the Medical Library Association: JMLA*, 105(3):285-289. doi: 10.5195/jmla.2017.183.

2. Clark JM, Sanders S, Carter M, Honeyman D, Cleo G, Auld Y, Booth D, Condron P, Dalais C, Bateup S, Linthwaite B, May N, Munn J, Ramsay L, Rickett K, Rutter C, Smith A, Sondergeld P, Wallin M, Jones M, Beller E. (2020) Improving the translation of search strategies using the Polyglot Search Translator: a randomized controlled trial. *Journal of the Medical Library Association: JMLA*. 108(2):195-207. doi: 10.5195/jmla.2020.834.

3. Bramer, W. M., Giustini, D., de Jonge, G. B., Holland, L., & Bekhuis, T. (2016). De-duplication of database search results for systematic reviews in EndNote. *Journal of the Medical Library Association: JMLA*, 104(3), 240-243. doi: 10.3163/1536-5050.104.3.014

1. Medline

Interface: Ovid MEDLINE(R) ALL<br><br>Date of Search: 14 March 2024<br><br>Number of hits: 2 398<br><br>Comment: In Ovid, two or more words are automatically searched as phrases; i.e. no quotation marks are needed	Field labels<br><br>• exp/ = exploded MeSH term<br>• / = non exploded MeSH term<br>• .ti,ab,kf. = title, abstract and author keywords<br>• adjx = within x words, regardless of order<br>• * = truncation of word for alternate endings

Database(s): **Ovid MEDLINE(R) ALL** 1946 to March 13, 2024
Search Strategy:

#	Searches	Results
1	COVID-19/	257196
2	exp COVID-19 Testing/	12295
3	COVID-19 Vaccines/	24034
4	SARS-CoV-2/	165903
5	(Coronavirus/ or Betacoronavirus/ or Coronavirus infections/) and (Disease outbreaks/ or Epidemics/ or Pandemics/)	40280
6	(nCoV* or 2019nCoV or 19nCoV or COVID* or SARS-COV-2 or SARSCOV-2 or SARS-COV2 or SARSCOV2 or SARS coronavirus 2 or Severe Acute Respiratory Syndrome Coronavirus 2 or Severe Acute Respiratory Syndrome Corona Virus 2).ti,ab,kf,nm,ox,rx,px.	402076
7	((coronavirus* or corona virus* or betacoronavirus*) and (pandemic* or epidemic* or outbreak* or crisis)).ti,ab,kf.	78954
8	((Wuhan or Hubei) adj5 pneumonia).ti,ab,kf.	427
9	((new or novel or "19" or "2019" or Wuhan or Hubei or China or Chinese) adj3 (coronavirus* or corona virus* or betacoronavirus* or CoV or HCoV)).ti,ab,kf.	100377
10	or/1-9	418067
11	Cost-Effectiveness Analysis/	786
12	Cost-Benefit Analysis/	94203
13	((cost? adj3 (benefit* or effect* or utilit* or minimi* or consequence*)) and analy*).ti,ab,kf.	100124
14	(economic adj3 evaluat*).ti,ab,kf.	19932
15	(cost-benefit* or cost-effectivenes* or cost-utility* or cost-minimi#ation* or cost-consequence*).ti,ab,kf.	95594
16	11 or 12 or 13 or 14 or 15	191149
17	10 and 16	2459
**18**	**limit 17 to (english language and yr="2019 -Current")**	**2398**

Karolinska Institutet

2. Embase

Interface: embase.com<br><br>Date of Search: 14 March 2024<br><br>Number of hits: 4 079<br><br>Comment: Emtree is the controlled vocabulary in Embase	Field labels<br><br>• /exp = exploded Emtree term<br>• /de = non exploded Emtree term<br>• ti,ab,kw = title, abstract and author keywords<br>• NEAR/x = within x words, regardless of order<br>• * = truncation of word for alternate endings

#	Searches	Results
#01	'coronavirus disease 2019'/exp	387373
#02	'covid-19 testing'/exp	11114
#03	'sars-cov-2 vaccine'/de	31732
#04	'severe acute respiratory syndrome coronavirus 2'/exp	110346
#05	('coronavirinae'/de OR 'betacoronavirus'/de OR 'coronavirus infection'/de) AND ('epidemic'/exp OR 'pandemic'/de)	11208
#06	ncov*:ti,ab,kw OR 2019ncov:ti,ab,kw OR 19ncov:ti,ab,kw OR covid*:ti,ab,kw OR 'sars cov 2':ti,ab,kw OR 'sarscov 2':ti,ab,kw OR 'sars cov2':ti,ab,kw OR sarscov2:ti,ab,kw OR 'sars coronavirus 2':ti,ab,kw OR 'severe acute respiratory syndrome coronavirus 2':ti,ab,kw OR 'severe acute respiratory syndrome corona virus 2':ti,ab,kw	456446
#07	(coronavirus*:ti,ab,kw OR 'corona virus*':ti,ab,kw OR betacoronavirus*:ti,ab,kw) AND (pandemic*:ti,ab,kw OR epidemic*:ti,ab,kw OR outbreak*:ti,ab,kw OR crisis:ti,ab,kw)	79831
#08	((wuhan OR hubei) NEAR/5 pneumonia):ti,ab,kw	523
#09	((new OR novel OR 19 OR 2019 OR wuhan OR hubei OR china OR chinese) NEAR/3 (coronavirus* OR 'corona virus*' OR betacoronavirus* OR cov OR hcov)):ti,ab,kw	104418
#10	#1 OR #2 OR #3 OR #4 OR #5 OR #6 OR #7 OR #8 OR #9	502646
#11	'economic evaluation'/exp	364127
#12	((cost$ NEAR/3 (benefit* OR effect* OR utilit* OR minimi* OR consequence*)):ti,ab,kw) AND analy*:ti,ab,kw AND ((economic NEAR/3 evaluat*):ti,ab,kw)	12924
#13	'cost benefit*':ti,ab,kw OR 'cost effectivenes*':ti,ab,kw OR 'cost utility*':ti,ab,kw OR 'cost minimi?ation*':ti,ab,kw OR 'cost consequence*':ti,ab,kw	137915
#14	(economic NEAR/3 evaluat*):ti,ab,kw	28285
#15	#11 OR #12 OR #13 OR #14	397206
#16	#10 AND #15	5382
#17	#10 AND #15 AND [english]/lim AND [2019-2023]/py	5073
#18	#10 AND #15 AND [english]/lim AND [2019-2023]/py AND ([conference abstract]/lim OR [conference paper]/lim OR [conference review]/lim)	994
**#19**	**#17 NOT #18**	**4079**

3. Cochrane Library

Interface: Wiley<br><br>Date of Search: 14 March 2024<br><br>Number of hits: 323	Field labels<br>• ti,ab,kw = title, abstract and author keywords<br>• NEAR/x = within x words, regardless of order<br>• * = truncation of word for alternate endings

#	Searches	Results
#1	[mh ^COVID-19]	7483
#2	[mh "COVID-19 Testing"]	131
#3	[mh ^"COVID-19 Vaccines"]	616
#4	[mh ^SARS-CoV-2]	3126
#5	([mh ^Coronavirus] OR [mh ^Betacoronavirus] OR [mh ^"Coronavirus infections"]) AND ([mh ^"Disease outbreaks"] OR [mh ^Epidemics] OR [mh ^Pandemics])	376
#6	(nCoV*:ti,ab,kw OR 2019nCoV:ti,ab,kw OR 19nCoV:ti,ab,kw OR COVID*:ti,ab,kw OR SARS-COV-2:ti,ab,kw OR SARSCOV-2:ti,ab,kw OR SARS-COV2:ti,ab,kw OR SARSCOV2:ti,ab,kw OR "SARS coronavirus 2":ti,ab,kw OR "Severe Acute Respiratory Syndrome Coronavirus 2":ti,ab,kw OR "Severe Acute Respiratory Syndrome Corona Virus 2":ti,ab,kw)	20450
#7	((coronavirus*:ti,ab,kw OR ("corona" NEXT virus*):ti,ab,kw OR betacoronavirus*:ti,ab,kw) AND (pandemic*:ti,ab,kw OR epidemic*:ti,ab,kw OR outbreak*:ti,ab,kw OR crisis:ti,ab,kw))	4451
#8	((Wuhan:ti,ab,kw OR Hubei:ti,ab,kw) NEAR/5 pneumonia:ti,ab,kw)	29
#9	((new:ti,ab,kw OR novel:ti,ab,kw OR 19:ti,ab,kw OR 2019:ti,ab,kw OR Wuhan:ti,ab,kw OR Hubei:ti,ab,kw OR China:ti,ab,kw OR Chinese:ti,ab,kw) NEAR/3 (coronavirus*:ti,ab,kw OR ("corona" NEXT virus*):ti,ab,kw OR betacoronavirus*:ti,ab,kw OR CoV:ti,ab,kw OR HCoV:ti,ab,kw))	9837
#10	#1 OR #2 OR #3 OR #4 OR #5 OR #6 OR #7 OR #8 OR #9	20981
#11	[mh ^"Cost-Effectiveness Analysis"]	103
#12	[mh ^"Cost-Benefit Analysis"]	11368
#13	((cost?:ti,ab,kw NEAR/3 (benefit*:ti,ab,kw OR effect*:ti,ab,kw OR utilit*:ti,ab,kw OR minimi*:ti,ab,kw OR consequence*:ti,ab,kw)) AND analy*:ti,ab,kw)	30847
#14	(economic:ti,ab,kw NEAR/3 evaluat*:ti,ab,kw)	5793
#15	(cost-benefit*:ti,ab,kw OR cost-effectivenes*:ti,ab,kw OR cost-utility*:ti,ab,kw OR cost-minimi?ation*:ti,ab,kw OR cost-consequence*:ti,ab,kw)	32461
#16	#11 OR #12 OR #13 OR #14 OR #15	38315
**#17**	**#10 AND #16 NOT (clinicaltrials or trialsearch):so**	**323**

4. Web of Science Core Collection

Interface: Clarivate Analytics<br><br>Editions = A&HCI , ESCI , SCI-EXPANDED , SSCI<br><br>Date of Search: 14 March 2024<br><br>Number of hits: 2 852	Field labels<br><br>• TS/Topic = title, abstract, author keywords and Keywords Plus<br>• NEAR/x = within x words, regardless of order<br>• * = truncation of word for alternate endings<br><br>Note: the *Exact search*-function was used for all the searches

#	Searches	Results
1	TS=(nCoV* OR 2019nCoV OR 19nCoV OR COVID* OR SARS-COV-2 OR SARSCOV-2 OR SARS-COV2 OR SARSCOV2 OR "SARS coronavirus 2" OR "Severe Acute Respiratory Syndrome Coronavirus 2" OR "Severe Acute Respiratory Syndrome Corona Virus 2")	520593
2	TS=((coronavirus* OR "corona virus*" OR betacoronavirus*) AND (pandemic* OR epidemic* OR outbreak* OR crisis))	95256
3	TS=((Wuhan OR Hubei) NEAR/4 pneumonia)	461
4	TS=((new OR novel OR 19 OR 2019 OR Wuhan OR Hubei OR China OR Chinese) NEAR/2 (coronavirus* OR "corona virus*" OR betacoronavirus* OR CoV OR HCoV))	106202
5	#4 OR #3 OR #2 OR #1	532699
6	TS=((cost$ NEAR/2 (benefit* OR effect* OR utilit* OR minimi* OR consequence*)) AND analy*)	182784
7	TS=(economic NEAR/2 evaluat*)	37381
8	TS=(cost-benefit* OR cost-effectivenes* OR cost-utility* OR cost-minimi?ation* OR cost-consequence*)	160818
9	#8 OR #7 OR #6	277367
10	#9 AND #5	2900
11	#9 AND #5 and 2019 or 2020 or 2021 or 2022 or 2023 or 2024 (Publication Years)	2884
**12**	**#9 AND #5 and 2019 or 2020 or 2021 or 2022 or 2023 or 2024 (Publication Years) and English (Languages)**	**2852**

Table S1. World Bank Economy Groups of economies

Economy Classification [a]	Gross national income (GNI) per capita (US$) [b]
Low-income economy (LIE)	≤ 1,135
Lower-middle-income economy (LMIE)	1,136 ~ 4,465
Upper-middle-income economy (UMIE)	4,466 ~ 13,845
High-income economy (HIE)	≥13,846

a. Fiscal year 2024
b. Fiscal year 2022

Table S2. Characteristics of the included studies

Author	Year	Country/Area	Economy[a]	Perspective	Time horizon	Discount rate[b]		Economic evaluation[c]	WTP	Population	Population size[d]	Intervention[e]	Comparator[f]	Measure of effectiveness[g]	INB (US$)[h]	SD of INB (US$)[i]	JBI score
						Cost	Utility										
Atherly[1]	2023	US	HI	Societal	2 years	NA	NA	CEA	73309 USD	General population	330 M	Hospital improvement	NI	QALY	926.99	141.89	8
												NMIs			-6677.38	1022.05	
												Vaccines			187.17	28.65	
												NMIs+Hospital improvement			-5119.98	783.67	
												NMIs+Vaccine			-6243.39	955.62	
												Hospital improvement+Vaccine			712.31	109.03	
												NMIs+Hospital improvement+Vaccine			-5342.65	817.75	
Augustovski[2]	2023	Argentina	UMI	Health sector	1 year	No	3%	CEA	10636 USD	General population	29.78 M	Vaccination campaign	NI	QALY	728.92	120.56	10
		Brazil	UMI						7697 USD		150.03 M				218.17	17.47	
		Chile	HI						16247 USD		13.45 M				319.18	52.19	
		Colombia	UMI						6183 USD		36.06 M				421.37	59.96	
		Costa Rica	UMI						12537 USD		3.56 M				548.25	86.34	
		Mexico	UMI						10046 USD		84.89 M				666.07	88.83	
		Peru	UMI						6635 USD		22.25 M				574.91	94.55	
Bagepally[3]	2021	India	LMI	Health sector	1 year	No	No	CEA	142719 INR	General population	1 M	Mask	NI	QALY	-3391.39	2767.67	10
												Hand hygiene			-119.29	23.36	
Bartsch[4]	2024	US	HI	Societal	3 months	NA	NA	CEA	50000 USD	Nursing home residents	100	Testing	NI	QALY	-990.24	335.27	7
				Health sector											-128.35	157.60	
Broughel[5]	2021	US	HI	Societal	NA	NA	5%	CBA	50000 USD	General population	329.5 M	Suppression	NI	QALY	1461.34	218.65	8
Cai[6]	2023	Singapore	HI	Societal	1 month	3%	3%	CEA	59798 USD	General population	5.454 M	Border control	NI	QALY	10.92	2.88	10
		Thailand	UMI						7189 USD		71.6 M				8.91	0.86	
Choi[7]	2023	South Korea	HI	Societal	1 year	NA	NA	CEA	34998 USD	General population	51.29 M	Enhanced vaccination	Vaccination	QALY	25.08	8.36	8
				Health sector											5.36	3.99	
Cook[8]	2021	Australia	HI	Health sector	1 year	No	No	CBA	NA	General population	2590290	Herd immunity	NI	VSL	1271.01	131.29	9
												Suppression			2729.98	398.51	
Debrabant[9]	2021	Denmark	HI	Health sector	6 months	NA	0-4%	CEA	398314 DKK	General population	5.831 M	Vaccine	NI	QALY	15.84	38.60	8
Di Fusco[10]	2023	US	HI	Payer	1 year	3%	3%	CEA	50000 USD	General population	310.184 M	Single booster	No booster	QALY	67.99	39.80	10

				Societal											110.25	41.60	
Drakesmith[11]	2022	UK	HI	Health sector	NA	No	1.5%	CEA	60000 GBP	General population	22021	Mass LFD testing	Normal testing	QALY	479.17	73.04	7
Du[12]	2023	India	LMI	Health sector	200 days	NA	3%	CEA	Varied	General population	1.366 B	Fractional vaccine booster	Standard vaccine booster	YLL	11.70	30.14	9
Du (1)[13]	2022	India	LMI	Health sector	NA	NA	3%	CEA	Varied	General population	1.366 B	Fractional vaccine	NI	YLL	52.24	52.35	8
Du (2)[14]	2022	US	HI	Direct societal	150 days	NA	3%	CEA	100000 USD	General population	328 M	Proactive testing	Normal testing	YLL	6502.69	2734.44	8
Du[15]	2021	US	HI	Health sector	150 days	NA	3%	CEA	100000 USD	General population	328.2 M	Expanding PCR testing	NI	YLL	6222.16	3747.91	9
Fernandes[16]	2022	Brazil	UMI	Health sector	1 year	No	No	CEA	17586 USD		NA	Vaccine	NI	QALY	16.58	16.76	7
Ferranna[17]	2023	US	HI	Payer	6 months	3%	3%	CBA	NA	Employees	332 M	Vaccine mandate	No vaccine mandate	QALY	410.00	214.68	8
Fu[18]	2022	China	UMI	Societal	1 year	No	No	CEA	12556 USD	General population	NA	Booster	No booster	QALY	1046.02	604.27	8
Fust[19]	2024	Japan	HI	Health sector	1 year	No	2%	CEA	5000000 JPY	General population	124.97 M	Enhanced vaccination	NI	QALY	121.61	38.12	8
				Societal											156.08	38.12	
Gandjour[20]	2022	Germany	HI	Societal	5 years	3%	2%	CEA	101493 USD	General population	NA	Vaccine	Partial lockdown	QALY	895.61	374.70	8
Gandjour[21]	2023	Germany	HI	Societal	1 and 5 years	3%	2%	CEA	101493 EUR	General population	NA	Lockdown+successful revaccination	NI	LY	2431.17	2505.85	8
Gonzalez Lopez-Valcarcel[22]	2021	Spain	HI	Health sector	2 years	3%	3%	CEA	25000 EUR	General population	46940000	TTQ	NI	QALY	6.08	2.25	7
Guzman Ruiz[23]	2022	Colombia	UMI	Societal	1 year	NA	3%	CEA	6436 USD	General population	50.3 M	TTI	NI	QALY	11334.13	1303.35	8
				Health sector											10764.07	1303.35	
Hagens[24]	2021	Turkey	UMI	Societal	1 year	3%	3%	CEA	9127 USD	General population	83429615	Vaccine	NI	QALY	649.48	36.19	8
				Health sector											425.21	23.68	
Hanig[25]	2023	US	HI	Societal	NA	NA	NA	CBA	Varied	General population	1.2 M	Improved public transportation	NI	VSL	-4.43	53.85	8
Heatley[26]	2022	New Zealand	HI	Societal	NA	No	No	CBA	73450 NZD	General population	5.124 M	Lockdown	Limited opening	QALY	-111.90	330.18	6
Jiang[27]	2022	Hong Kong	HI	Societal	1 year	3%	3%	CEA	49800 USD	General population	100000	Vaccine	NI	QALY	639.64	208.55	10
		Indonesia	UMI						4332 USD						173.20	74.64	
		China	UMI						12556 USD						146.03	58.31	
		Philippines	LMI						3460 USD						295.59	156.72	
		Singapore	HI						72794 USD						441.05	212.45	
		Thailand	UMI						7066 USD						238.57	94.26	
Joshi[28]	2024	Germany	HI	Health sector	1 year	No	3%	CEA	50000 USD	General population	83.329 M	Enhanced vaccination	Vaccine	QALY	76.06	2.06	9
Karnon[29]	2022	Australia	HI	Societal	6 months	NA	NA	CBA	50000 AUD	General population	26.26 M	Testing	NI	QALY	7947.66	8677.55	7
Kirson[30]	2022	US	HI	Societal	3.5 years	3%	3%	CBA	150000 USD	General population	330 M	Vaccine	NI	LY	15151.50	7606.68	7

Kirwin[31]	2021	Canada	HI	Payer	NA	1.5%	1.5%	Net benefit	30000 CAD	General population	4.371 M	Vaccine	NI	QALY	52.47	26.67	8
Kohli[32]	2023	US	HI	Health sector	1 year	No	3%	CEA	81630 USD	General population	335.942	Enhanced vaccination	NI	QALY	125.44	18.83	10
				Societal											160.14	18.83	
Kohli (1)[33]	2021	US	HI	Health sector	1 year	NA	3.0%	CEA	50000 USD	General population	326.8 M	Vaccine	NI	QALY	106.08	24.91	9
Kohli (2)[34]	2021	UK	HI	Health sector	10 years	3.5%	3.5%	CEA	25000 GBP	General population	66.84 M	Expanding vaccine	Vaccine	QALY	4.62	0.87	8
				Societal											7.94	0.87	
Li[35]	2024	US	HI	Health sector	180 days	1.5%	1.5%	CEA	50000 USD	General population	331.89 M	Enhanced vaccination	NI	QALY	16.01	9.22	9
Li[36]	2023	Australia	HI	Health sector	180 days	3%	3%	CEA	50000 AUD	General population	26 M	Vaccine booster	Vaccine	QALY	25.63	12.49	9
Li[37]	2022	US	HI	Health sector	180 days	3%	3%	CEA	50000 USD	General population	100000	Vaccine booster	Vaccine	QALY	33.20	59.70	8
Lopez Segui (1)[38]	2021	Spain	HI	Health sector	NA	NA	3%	CBA	25000 EUR	General population	124865	Mass testing	Normal testing	QALY	54.92	13.41	8
Lopez Segui (2)[39]	2021	Spain	HI	Societal	NA	No	No	CBA	25000 EUR	General population	1986032	Vaccine	NI	QALY	298.96	86.74	8
				Health sector											51.07	26.08	
Lou[40]	2023	Singapore	HI	Societal	1 month	No	3%	CBA	75000 USD	General population	5.64 M	Testing+quarantine for travelers	NI	QALY	68.45	115.97	9
												Quarantine for travelers			208.99	248.76	
Mar[41]	2024	Spain	HI	Health sector	6 months	NA	3%	CEA	25000 EUR	General population	2435495	Vaccine	NI	QALY	232.88	76.71	9
Marco-Franco[42]	2021	Spain	HI	Buyer	NS	NA	3-4%	CEA	22000-33000 EUR	General population	47.37 M	Vaccine	NI	QALY	4571.67	399.75	8
Maya[43]	2022	US	HI	Societal	NA	NA	3%	CEA	70219 USD	School student	1000	PCR and/or Antigen test	NI	QALY	-8.68	5.73	8
Miles[44]	2021	UK	HI	Societal	NA	NA	NA	CBA	30000 GBP	General population	67.1 M	Lockdown	NI	QALY	-8239.32	3336.85	7
Mol[45]	2023	Denmark, Sweden	HI	Societal	6 months	No	NA	CEA	100000 USD	General population	5.8 M, 10.1 M	Lockdown	NI	LY	-214.48	1826.45	8
Morales-Zamora[46]	2022	Colombia	UMI	Health sector	3 years	No	No	CEA	5304.3 USD	General population	50.93 M	Vaccine	NI	DALY	4.60	33.08	9
Neilan[47]	2021	US	HI	Health sector	180 days	No	3%	CEA	100000 USD	General population	6.9 M	Expanding PCR testing	Hospitalized testing	QALY	317.96	313.31	8
Nguyen[48]	2022	Australia	HI	Societal	118 days	No	No	CEA	Varied	General population	2393	Social distancing	No social distancing	DALY	1083.97	1185.79	8
Nurchis[49]	2022	Italy	HI	Societal	3 months	No	3%	CBA	NA	Healthcare worker	5152	Vaccination campaign	NI	PL	2779.16	51.73	8
Orangi[50]	2022	Kenya	LMI	Societal	1.5 years	3%	3%	CEA	3000 USD	General population	47.56 M	Vaccine	no vaccine	DALY	31.37	4.29	9
Orlewska[51]	2022	Poland	HI	Payer	1 year	NA	3.5%	CEA	147024 PLN	General population	37.75 M	Vaccine	no vaccine	QALY	1391.29	53.28	8
Padula[52]	2021	US	HI	Health sector	1 year	NA	NA	CEA	100000 USD	General population	330 M	Vaccine	No vaccine	QALY	2279.25	125.04	8
Patenaude[53]	2022	US	HI	Health sector	78 days	NA	NA	CBA	NA	General population	331.9 M	Oral vaccine	Vaccine	Case averted	1.40	0.27	8
				Societal											127.61	357.77	
Pearson[54]	2021	Pakistan	LMI	Health sector	10 years	3%	3%	CEA	1322 USD	General population	48 M	Vaccine	NI	DALY	6.94	10.13	10
Reddy (1)[55]	2021	South Africa	UMI	Health sector	1 year	No	No	CEA	7055 USD	General population	59 M	Vaccine	NI	YLL	358.83	144.77	8

Reddy (2)[56]	2021	South Africa	UMI	Health sector	1 year	No	3%	CEA	3250 USD	General population	11 M	Testing+Contact tracing	Testing alone	LY	155.07	72.10	8
Ruiz[57]	2023	Nigeria	LMI	Health sector	1 year	3%	3%	CEA	2097 USD	General population	107.1 M	Vaccine	No vaccine	DALY	3.59	24.72	10
Sandmann[58]	2021	UK	HI	Health sector	10 years	3.5%	3.5%	CEA	20000 GBP	General population	67 M	Vaccine+physical distancing	No intervention	QALY	2204.55	1876.68	8
Scherbina[59]	2021	US	HI	Societal	NA	NA	3%	CBA	150000 USD	General population	332 M	Lockdown	No lockdown	QALY	570.67	492.14	7
Schonberger[60]	2020	US	HI	Societal	11.1 months	3%	3%	CBA	125000 USD	General population	329 M	Limited opening	Full opening	QALY	2918.82	426.07	9
Sirison[61]	2023	Thailand	UMI	Provider	170 days	3%	3%	CEA	1359 USD	General population	50 M	Vaccine booster	No booster	Case averted	76.94	1.56	7
Suwantika[62]	2022	Indonesia	UMI	Societal	1 year	NA	NA	CEA	NA	General population	271.6 M	Social distancing	NI	Death averted	6501.05	1886.51	8
Szanyi[63]	2023	Australia	HI	Societal	1 year	3%	3%	CEA	70000 AUD	General population	6.6 M	Vaccine booster	No booster	HALY	75.39	41.57	8
				Health sector											9.79	16.49	
Tan[64]	2023	China	HI*	Societal	82 days	NA	NA	CEA	173630 CNY	General population	24.89 M	Dynamic zero	NI	DALY	5255.50	493.14	10
													Lockdown		684.63	471.23	
Tuite[65]	2024	Canada	HI	Societal	Lifetime	1.5%	1.5%	CBA	30000 CAD	General population	38.45 M	Vaccine	NI	QALY	6760.74	2704.12	10
Vaezi[66]	2021	Iran	LMI	Health sector	NA	NA	NA	CEA	2282 USD	General population	79.9 M	Vaccine	NI	DALY	2.91	2.00	6
Viches[67]	2022	Canada	HI	Societal	1 year	1.5%	1.5%	CEA	30000 CAD	People at workplace	100,000	RA screening in workplaces	TOSC	QALY	-5.09	91.33	8
				Health sector											9.75	57.60	
Wang W[68]	2021	Taiwan	HI	Societal	180 days	No	No	CUA	28707 USD	General population	8362864	Vaccine	NI	QALD	689.81	120.99	8
Wang X[69]	2022	China	UMI	Provider	NA	No	No	CEA	12556 USD	Audience of competition events	5000	close-contact control	NI	Infection averted	14526.75	15452.44	8
Wang Y[70]	2022	Thailand	UMI	Societal	1 year	NA	NA	CEA	160000 THB	General population	71.6 M	Vaccine	NI	LY	177.36	207.01	8
Xiong[71]	2022	Hong Kong	HI	Health sector	1 year	NA	NA	CEA	1000000 HKD	General population	7346100	Vaccine	NI	QALY	266.11	271.50	8
Zafari[72]	2022	US	HI	Societal	1 year	3%	3%	CEA	63530 USD	Customers in restaurants	30	Improving ventilation	NI	QALY	8581.98	4302.44	9
Zafari[73]	2021	US	HI	Societal	90 days	No	3%	CEA	200000 USD	University students and staff	20500	Symptom-checking app	NI	QALY	55.94	72.98	8
												Thermal camera			-76.95	42.95	
												Gateway testing			183.66	257.46	
												Standardized masks			461.11	514.91	
												Weekly testing			-79.63	301.81	
												Package intervention			98.52	61.97	
Zhao[74]	2021	China	UMI	Health sector	60 days	NA	3%	CEA	70892 CNY	General population	1.336 B	Movement restriction	NI	DALY	2669.27	3541.67	10
				Societal											20976.23	19793.70	
Zhou[75]	2023	China	UMI	Societal	1 year	5%	5%	CUA	72000 CNY	General population	1.33 B	Vaccination	NI	QALY	18628.56	20310.31	11

a. HI, hi-income; LMI, lower-middle-income; UMI, upper-middle-income
b. NA, not available
c. CEA, cost-effectiveness analysis; CBA, cost-benefit analysis; CUA, cost-utility analysis
d. M, million; B, billion
e. NMIs, non-medical interventions; TTQ, test-tracking-quarantine; TTI, test, trace, and isolate
f. NI, null intervention; TOSC, RT-PCR testing of only severe symptomatic cases
g. QALY, quality-adjusted life year; VSL, value of statistical life; YLL, year of life lost; LY, life year; DALY, disability-adjusted life year; PL, productivity loss; HALY, health-adjusted life year; QALD, quality-adjusted life day
h. INB, incremental net benefit, adjusted for purchasing power parity
i. SD, standard deviation

* The study was conducted in Shanghai, China, therefore the economy group was assigned as high-income.

Table S3. Definitions of the preventive COVID-19 interventions compared in the included studies

Intervention	Definition
Border control	Repeated testing and necessary quarantine were implemented for cross-border travelers to minimize the importation of cases into the local community.[6]
Close-contact control	Increasing nucleic acid testing (NAT) frequency among sports competition participants. If an infection was detected, the affected individual was quarantined, and the entire sports delegation associated with the quarantined case underwent additional testing.[69]
Dynamic zero	A strategy implemented by Shanghai, China during the COVID-19 pandemic aimed to quickly identify and isolate COVID-19 cases. This approach was flexible, allowing for short-term increases in cases but emphasizing rapid response measures to control outbreaks. Key tactics included mass testing, stringent quarantine and isolation protocols, travel restrictions, localized lockdowns, and the use of technology for contact tracing and movement control.[64,76]
Enhanced vaccination	Including provide further vaccination after Omicron BA.1/2 variant[63], booster vaccination [10,36,37,61] (including fractional dosing booster[12]), vaccine mandates[17], vaccination campaign[2,49], sequential vaccination[18], expanding influenza vaccination[34],
Entry testing	See “Gateway testing”.
Expanding testing	The method to increase the scope or frequency of the COVID-19 testing, including surveillance testing[15], proactive testing[14], whole-area (or mass) testing[11,38],
Expanding influenza vaccination	Continuing to vaccinate all in age group 50 to 64 years with a quadrivalent cell-based vaccine (QIVc).[34]
Fractional dosing booster	Administering a lower dose (1/8~1/2) of a vaccine as a booster shot compared to the original full dose given during the initial vaccination series.[12]
Gateway testing	Testing individuals for the virus as they enter a specific area, facility, or country, designed to identify infected persons at points of entry—such as airports, borders, universities, workplaces, or events—to prevent them from introducing the virus into the new environment.[73]
Hand hygiene	The practice of regularly cleaning one’s hands to prevent the spread of the coronavirus.[3]
Herd immunity	It occurs when large portion of a community becomes immune to a disease, making the spread of the disease from person to person unlikely.[8]
Heterologous booster	Administering a booster dose of a COVID-19 vaccine that is different from the vaccine used for the initial vaccination series.[18]
Homogeneous booster	Administering a booster dose of a COVID-19 vaccine that is the same type as the vaccine used in the initial vaccination series.[18]
Hospital improvement (HI)	Improvement in hospital therapeutics and care.[1]
Hospitalized testing	Polymerase chain reaction (PCR) testing only for patients with severe/critical symptoms warranting hospitalization.[47]
Improved public transportation	To prevent crowding, transportation companies deploy autonomous vehicles, add extra buses, or utilize longer buses.[25]
Improving ventilation	Improving the indoor air ventilation using standalone filtration systems containing high efficiency particulate air or electrostatic units in poorly ventilated indoor commercial spaces.[72]
Limited opening	The phased and controlled reopening of businesses, services, and public spaces following full or partial lockdowns in the US during the COVID-19 pandemic.[60]
Lockdown	Imposition of strict restrictions on movement, social interactions, and economic activities by authorities to control the spread of the coronavirus. It typically involves closing non-essential businesses, restricting gatherings, halting public events, and limiting or completely stopping people's movement except for essential purposes such as healthcare, groceries, or work deemed essential.[21,44,59] Partial lockdown[20], and strict dynamic zero[64] were treated as lockdown in current study
Masks	Using surgical or N95 or standardized high-filtration masks to reduce the risk of infectious droplets spreading from person to person.[3,73]

Mass testing	Testing all individuals within a specific geographic area or community for COVID-19, regardless of whether they are showing symptoms of the disease.
Movement restriction	Measures implemented by governments and health authorities to limit the movement of people within and between areas to control the spread of the virus, often including travel bans, quarantine and isolation orders, social distancing, lockdowns, etc.[74]
Null intervention (NI)	The comparator group did not adopt any intervention or a specified intervention; however, general preventive measures as suggested locally or recommended by the World Health Organization were possibly adopted.
Nonmedical interventions (NMIs)	Measures to limit the physical contact of individuals (such as stay-at-home orders, the closing of schools, cancellation of public events, restrictions on social gatherings, closing public transportation, travel restrictions, and closing of nonessential businesses) and to reduce the probability of transmission per individual (such as wearing masks in public, tracking and tracing, and increased testing)[1], including package intervention[73].
Oral vaccine	An experimental oral vaccine against COVID-19, developed using the Vector-Adjuvant-Antigen Standardized Technology (VAAST) platform.[53]
Package intervention	A package of common interventions for the prevention of transmission of SARS-CoV-2 virus, excluding vaccines.[73]
Partial lockdown	A set of restrictive measures that are less severe than a full lockdown, aimed at slowing the spread of COVID-19, while allowing certain economic and social activities to continue under controlled conditions.[20]
Proactive testing	The strategy of actively testing individuals for the virus regardless of whether they show symptoms.[14]
Quarantine	Separating and restricting the movement of traveler across the border for a given period.[40]
Screening in workplaces	Implementation of frequent rapid antigen testing to detect asymptomatic infection in workplaces.[67]
Sequential vaccination	Administering a booster with a homogeneous booster or a heterogeneous booster.[18]
Social distancing	Maintaining a physical distance between individuals to reduce the spread of the virus. Recommendations typically included staying at least 1.5~2 meters away from others, avoiding large gatherings, and minimizing social interactions outside of one's household.[48]
Suppression	Providing police and other services jurisdiction to enforce quarantine and self-isolation measures to contain spread.[8]
Surveillance testing	In contrast to symptom-based testing and isolation, this approach involves testing all individuals regularly at specified intervals, accompanied by a period of isolation for those who test positive.[15]
Symptom-checking app	A digital tool available on smartphones and tablets that allows users to input their health information and symptoms to assess their risk of having contracted the COVID-19 virus.[73]
Testing	Using molecular tests or antigen tests to determine if an individual was infected with the SARS-CoV-2 virus, including those without synpmtom.[43,73]
Thermal camera	The cameras equipped with infrared technology to detect elevated body temperatures in individuals as part of efforts to identify possible infections.[73]
Testing of only severe symptomatic cases (TOSC)	Test only severe symptomatic cases. RT-PCR testing was implemented exclusively for cases exhibiting severe symptoms.[67]
Vaccination	Administering mRNA[39,55,68,71], viral vector[39,55], protein subunit, or inactivated/killed[27] vaccines to stimulate the immune system to protect against SARS-CoV-2 virus causing COVID-19.
Test, trace, and isolate/quarantine (TTI/TTQ)	Test, trace, and isolate/quarantine, including close-contact control[69]. The deployment of testing, contact tracing, isolation/quarantine, and monitoring for cases and their contacts. Once an index case is identified, patients must isolate with their cohabitant contacts. The index case must be monitored for assessment of warning signs or symptoms. Additionally, their close contacts in given days are traced, contacted, tested, and isolated/quarantined.[22,23,40,56]
Whole-area testing	See “Mass testing”[11]

Figure S1. Comparative cost-effectiveness of preventive COVID-19 interventions vs. NI from a societal perspective. **Panel A.** $INB_{ppp\text{-}adjusted}$. **Panel B.** $INB_{WTP\text{-}standardised}$. CI – confidence interval; HI – hospital improvement; INB – incremental net benefit; MD – mean difference; NI – null intervention; NMIs – nonmedical interventions; PPP – purchasing power parity; TTI – test, trace, and isolate; WTP – willingness to pay.

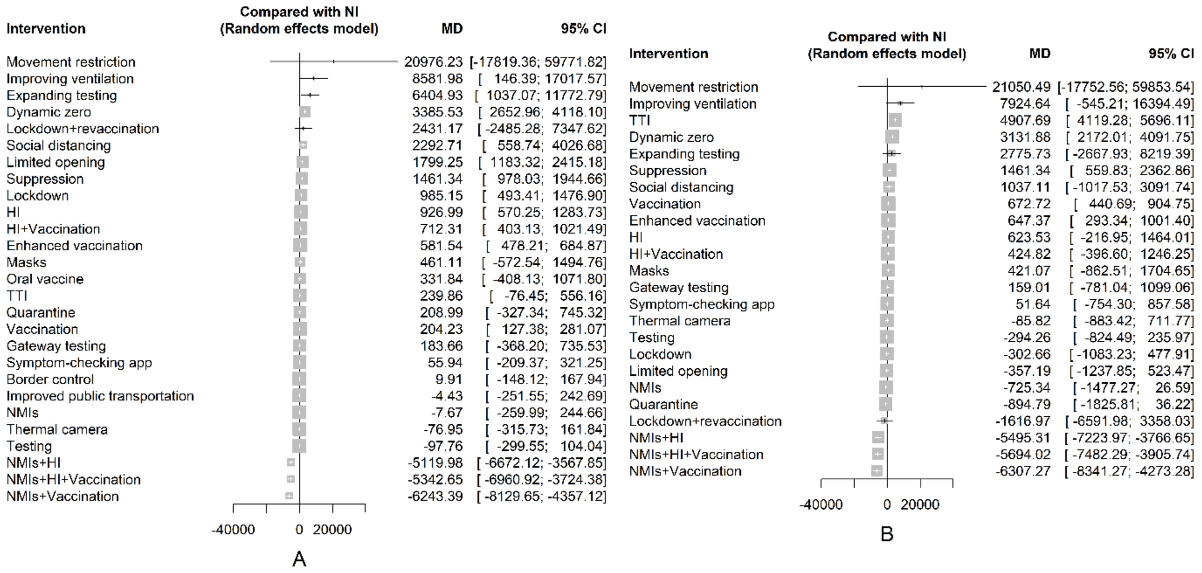

Figure S2. Interventions ranked by certainty of being superior across all comparisons from a societal perspective. **Panel A.** $INB_{ppp\text{-}adjusted}$. **Panel B.** $INB_{WTP\text{-}standardised}$. HI – hospital improvement; INB – incremental net benefit; NMIs – nonmedical interventions; PPP – purchasing power parity; SUCRA – surface under the cumulative ranking curve; TTI – test, trace, and isolate; WTP – willingness to pay.

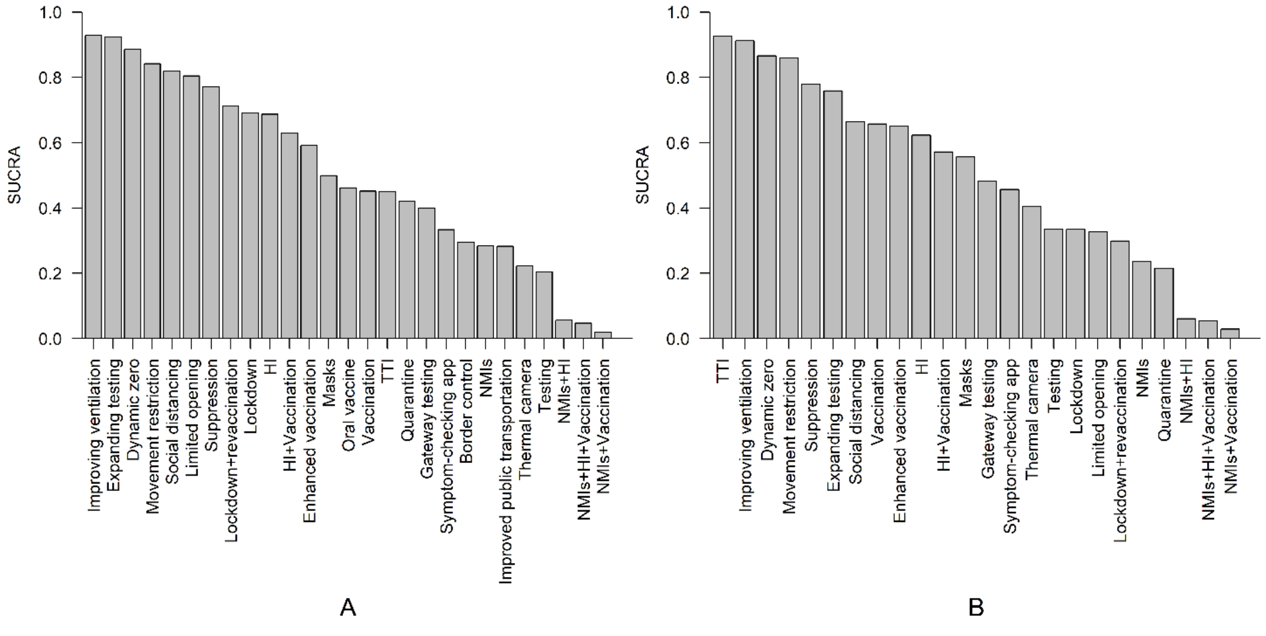

Figure S3. Comparative cost-effectiveness and ranks of the interventions vs. NI following study exclusion from a societal perspective. **Panel A.** Forest plot for $\text{INB}_{\text{WTP-standardised}}$. **Panel B.** Rank plot for $\text{INB}_{\text{WTP-standardised}}$. CI – confidence interval; HI – hospital improvement; INB – incremental net benefit; MD – mean difference; NI – null intervention; NMIs – nonmedical interventions; PPP – purchasing power parity; SUCRA – surface under the cumulative ranking curve; TTI – test, trace, and isolate; WTP – willingness to pay.

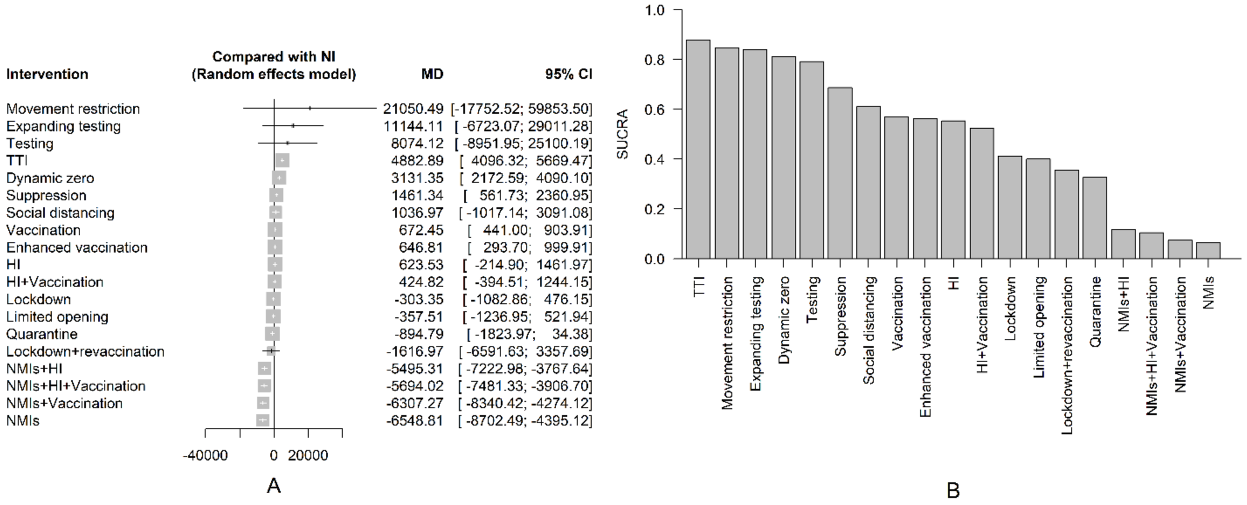

Figure S4. Split effects for highlighted comparisons in the network of societal perspective. **Panel A.** $\text{INB}_{\text{ppp-adjusted}}$. **Panel B.** $\text{INB}_{\text{WTP-standardised}}$. CI – confidence interval; INB – incremental net benefit; MD – mean difference; NI – null intervention; PPP – purchasing power parity; TTI – test, trace, and isolate; WTP – willingness to pay.

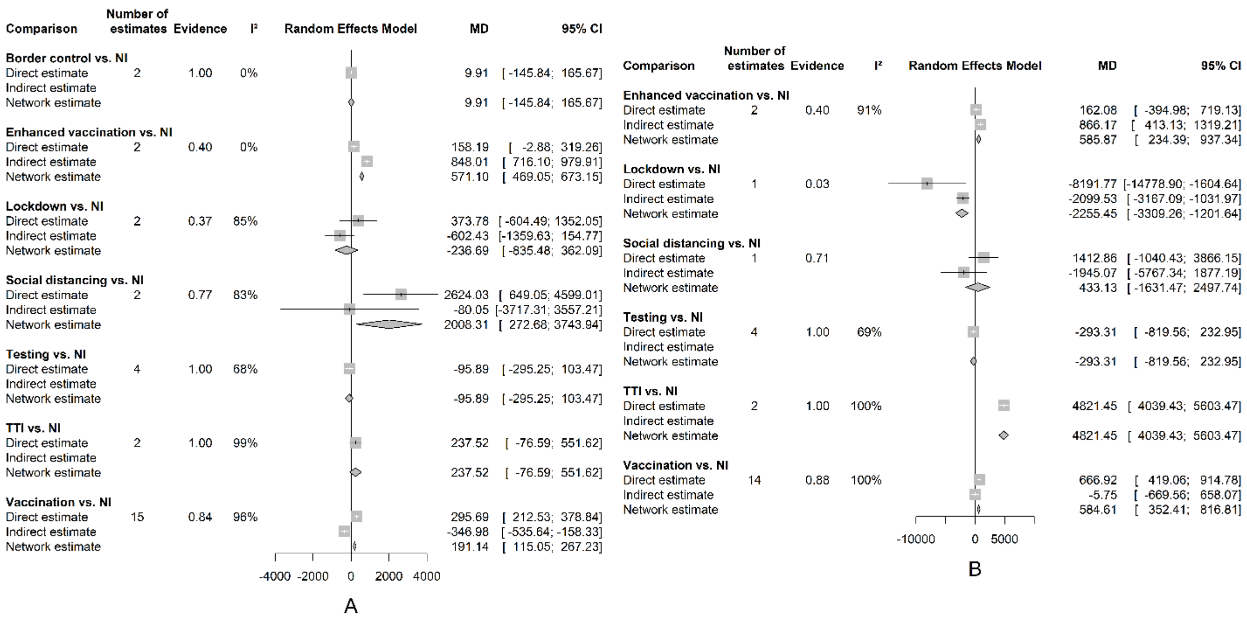

Figure S5. Split effects plot of the interventions in the constrained network of societal perspective. **Panel A.** $INB_{ppp\text{-}adjusted}$. **Panel B.** $INB_{WTP\text{-}standardised}$.

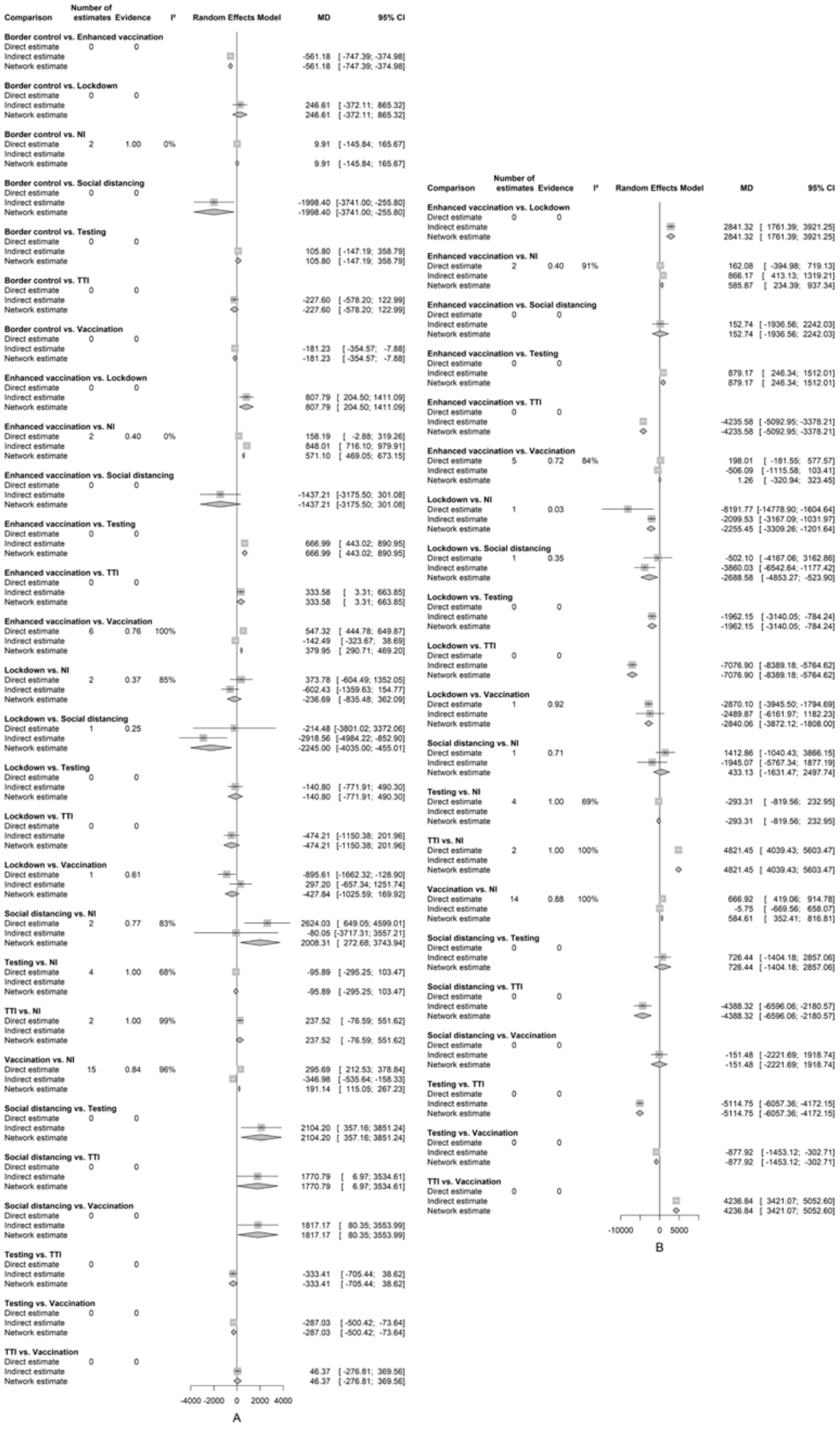

Figure S6. Evidence plot of the interventions in the constrained network of societal perspective

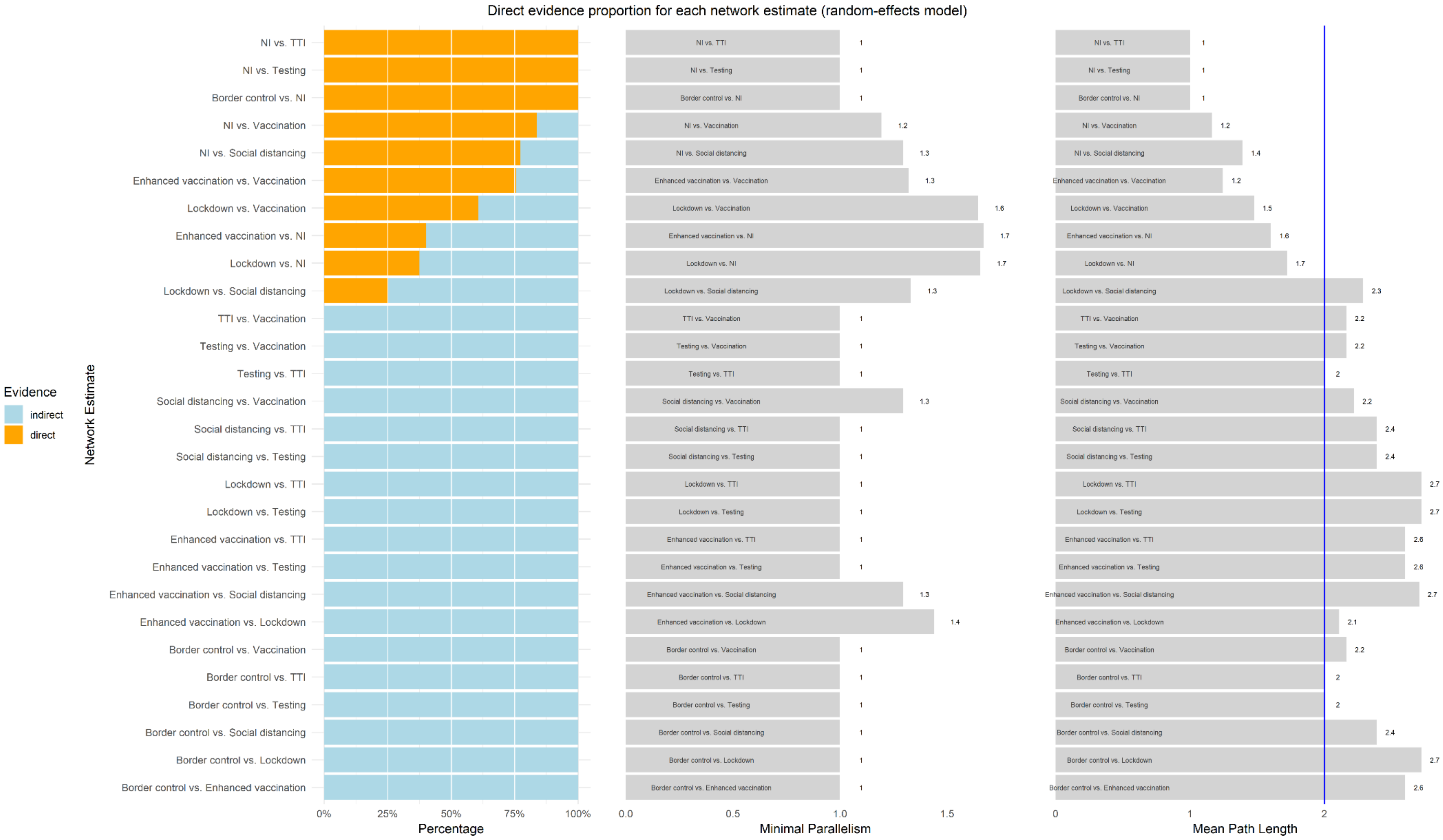

Figure S7. Funnel plots of comparisons among preventive COVID-19 interventions from a healthcare system perspective. **Panel A.** $INB_{ppp\text{-}adjusted}$. **Panel B.** $INB_{WTP\text{-}standardised}$. INB – incremental net benefit; PPP – purchasing power parity; WTP – willingness to pay.

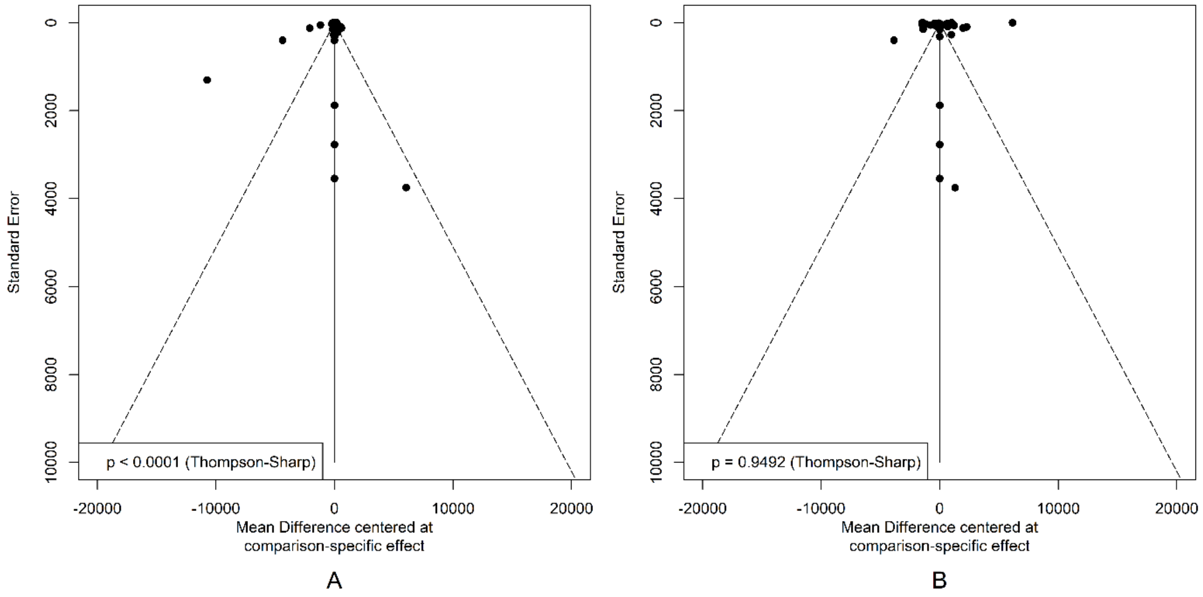

Figure S8. Comparative cost-effectiveness of preventive COVID-19 interventions vs. NI from a healthcare system perspective. **Panel A.** $INB_{ppp\text{-}adjusted}$. **Panel B.** $INB_{WTP\text{-}standardised}$. CI – confidence interval; HI – hospital improvement; INB – incremental net benefit; MD – mean difference; NI – null intervention; NMIs – nonmedical interventions; PPP – purchasing power parity; TTI – test, trace, and isolate; WTP – willingness to pay.

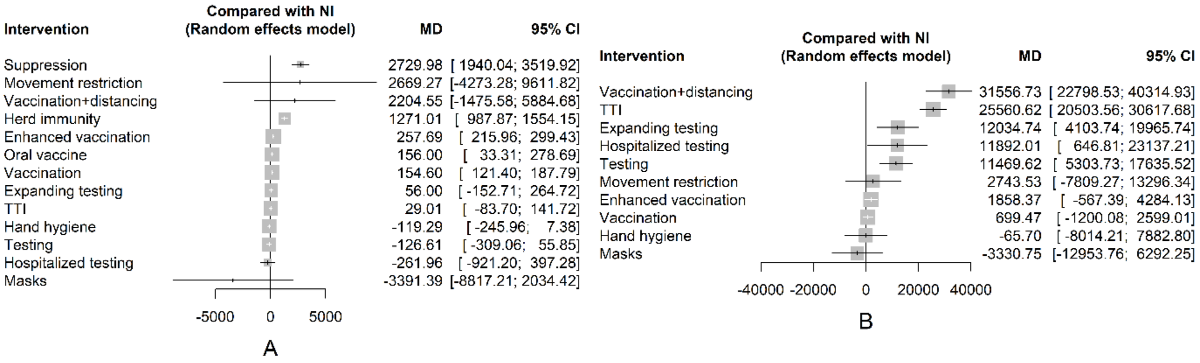

Figure S9. Interventions ranked by certainty of being superior across all comparisons from a healthcare system perspective. **Panel A.** $INB_{ppp\text{-}adjusted}$. **Panel B.** $INB_{WTP\text{-}standardised}$. INB – incremental net benefit; PPP – purchasing power parity; SUCRA – surface under the cumulative ranking curve; TTI – test, trace, and isolate; WTP – willingness to pay.

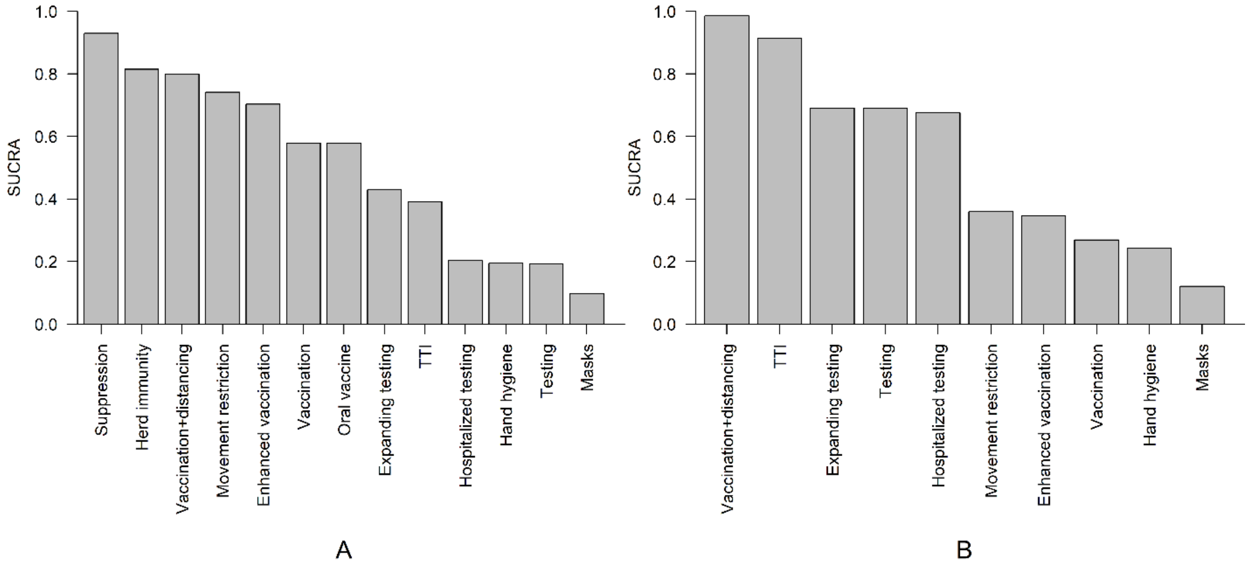

Figure S10. Comparative cost-effectiveness and ranks of the interventions vs. NI following study exclusion, from a healthcare system perspective. **Panel A.** Forest plot for $INB_{WTP\text{-}standardised}$. **Panel B.** Rank plot for $INB_{WTP\text{-}standardised}$. CI – confidence interval; INB – incremental net benefit; MD – mean difference; NI – null intervention; PPP – purchasing power parity; SUCRA – surface under the cumulative ranking curve; TTI – test, trace, and isolate; WTP – willingness to pay.

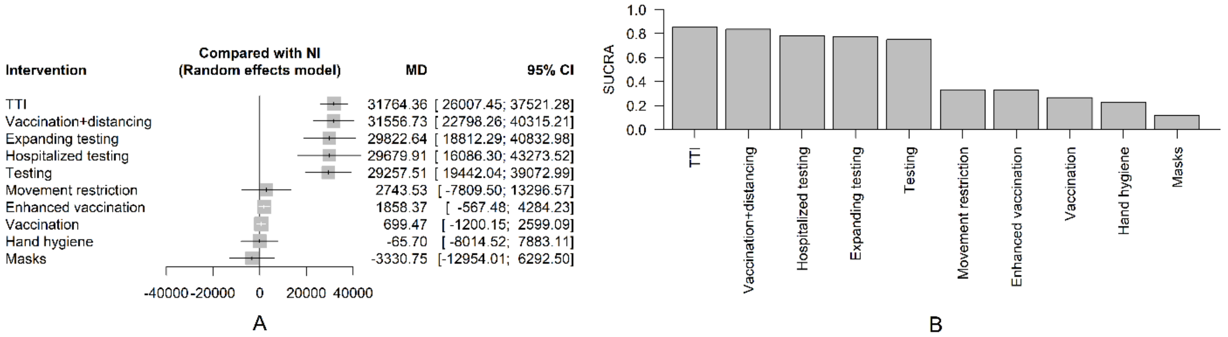

Figure S11. Split effects for highlighted comparisons in the network of healthcare system perspective. **Panel A.** $INB_{ppp\text{-}adjusted}$. **Panel B.** $INB_{WTP\text{-}standardised}$. CI – confidence interval; INB – incremental net benefit; MD – mean difference; NI – null intervention; PPP – purchasing power parity; TTI – test, trace, and isolate; WTP – willingness to pay.

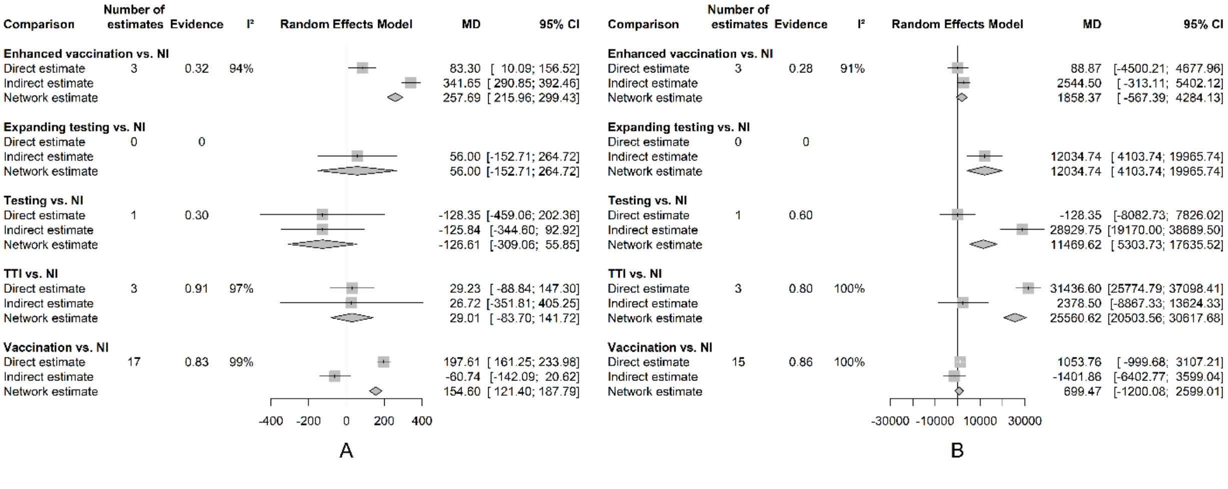

Figure S12. Split effects plot of the interventions in the constrained network of healthcare system perspective. **Panel A.** $INB_{ppp\text{-}adjusted}$. **Panel B.** $INB_{WTP\text{-}standardised}$. CI – confidence interval; MD – mean difference; NI – null intervention; PPP – purchasing power parity; TTI – test, trace, and isolate; WTP – willingness to pay.

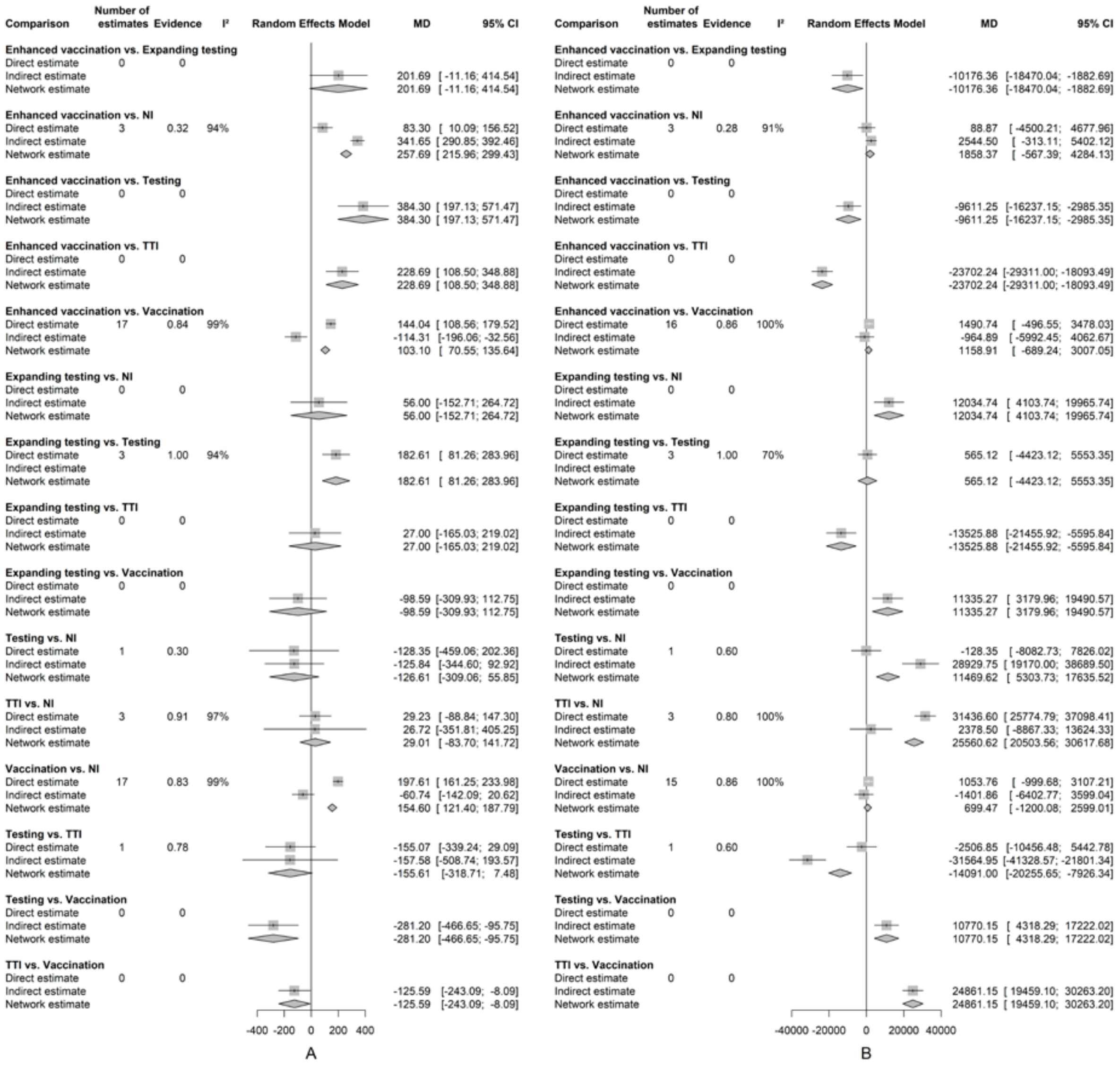

.

Figure S13. Evidence plot of the interventions in the constrained network of healthcare system perspective. NI – null intervention; PPP – purchasing power parity; TTI – test, trace, and isolate; WTP – willingness to pay.

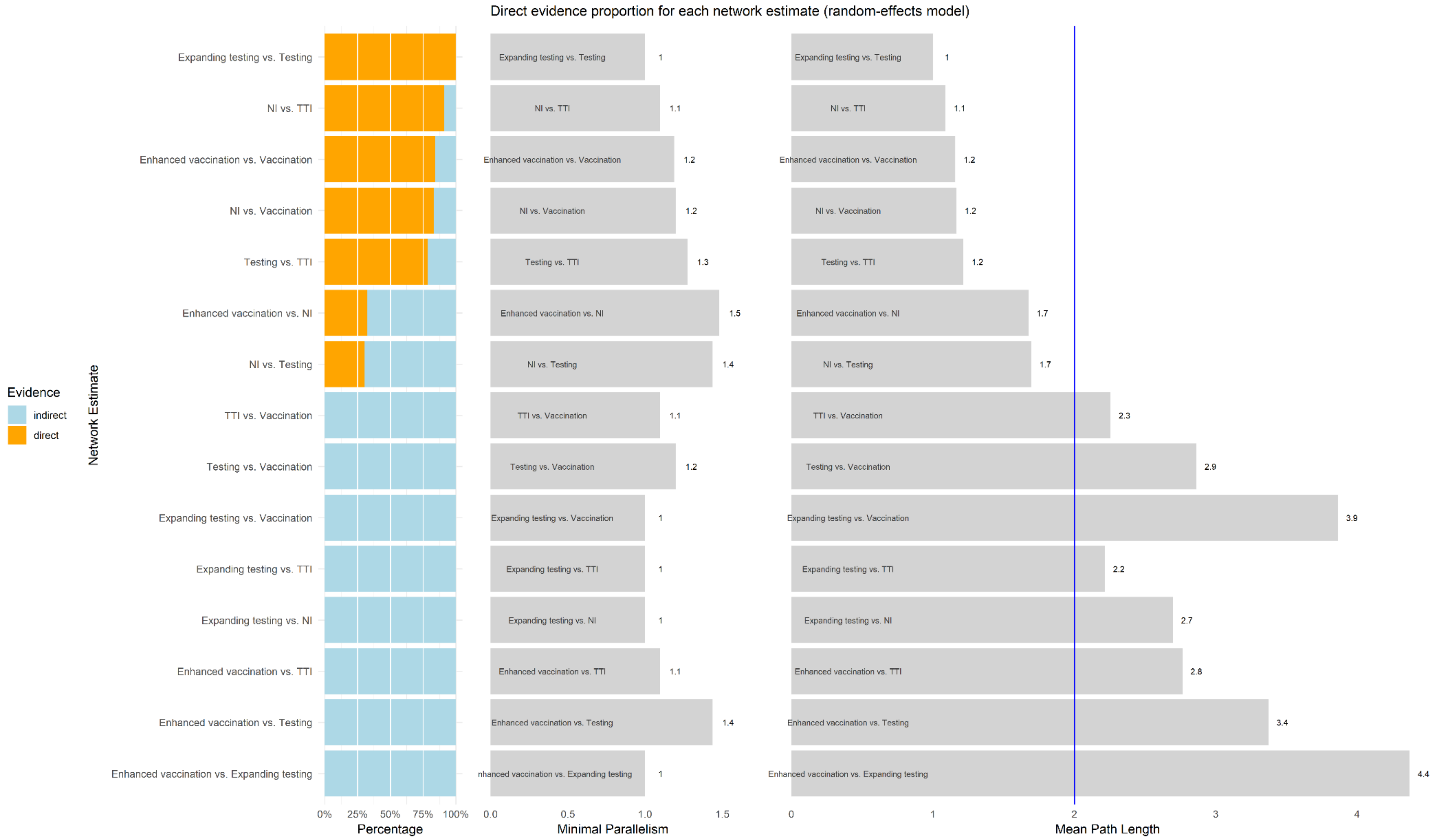

Figure S14. Subgroup analysis by economy levels, societal perspective. **Panel A.** $INB_{ppp\text{-}adjusted}$ (HIEs). **Panel B.** $INB_{WTP\text{-}standardized}$ (HIEs). **Panel C.** $INB_{ppp\text{-}adjusted}$ (UMIEs). **Panel D.** $INB_{WTP\text{-}standardized}$ (UMIEs). **Panel E.** $INB_{ppp\text{-}adjusted}$ (LMIEs). **Panel F.** $INB_{WTP\text{-}standardized}$ (LMIEs). CI – confidence interval; HI – hospital improvement; HIEs – high-income economies; LMIEs – lower-middle-income economies; MD – mean difference; NI – null intervention; NMIs – nonmedical interventions; TTI – test, trace, and isolate; UMIEs – upper-middle-income economies

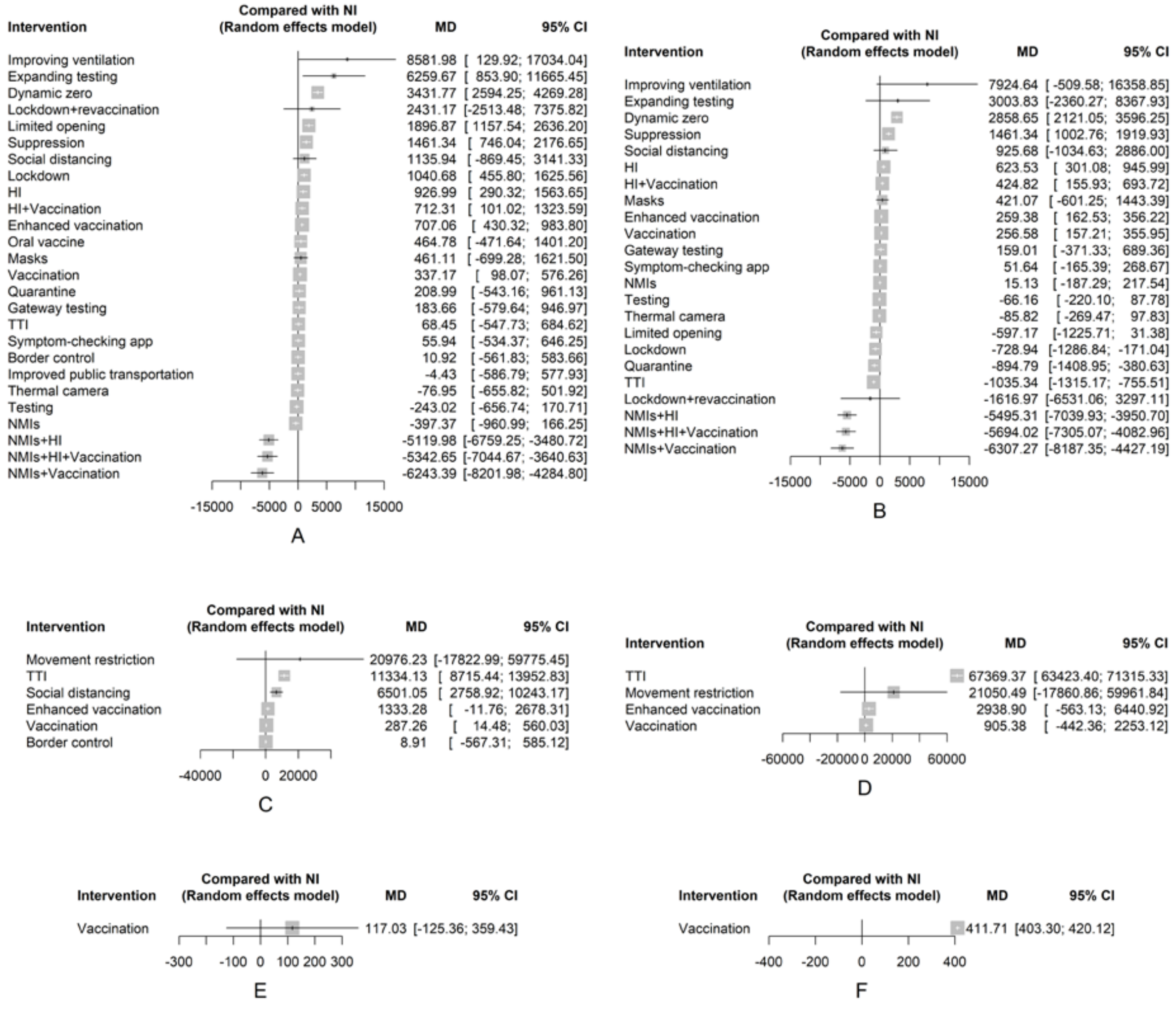

Figure S15. Subgroup analysis by economy levels, healthcare system perspective. **Panel A.** $INB_{ppp\text{-}adjusted}$ (HIEs). **Panel B.** $INB_{WTP\text{-}standardized}$ (HIEs). **Panel C.** $INB_{ppp\text{-}adjusted}$ (UMIEs). **Panel D.** $INB_{WTP\text{-}standardized}$ (UMIEs). **Panel E.** $INB_{ppp\text{-}adjusted}$ (LMIEs). **Panel F.** $INB_{WTP\text{-}standardized}$ (LMIEs). CI – confidence interval; HIEs – high-income economies; LMIEs – lower-middle-income economies; MD – mean difference; NI – null intervention; TTI – test, trace, and isolate; UMIEs – upper-middle-income economies

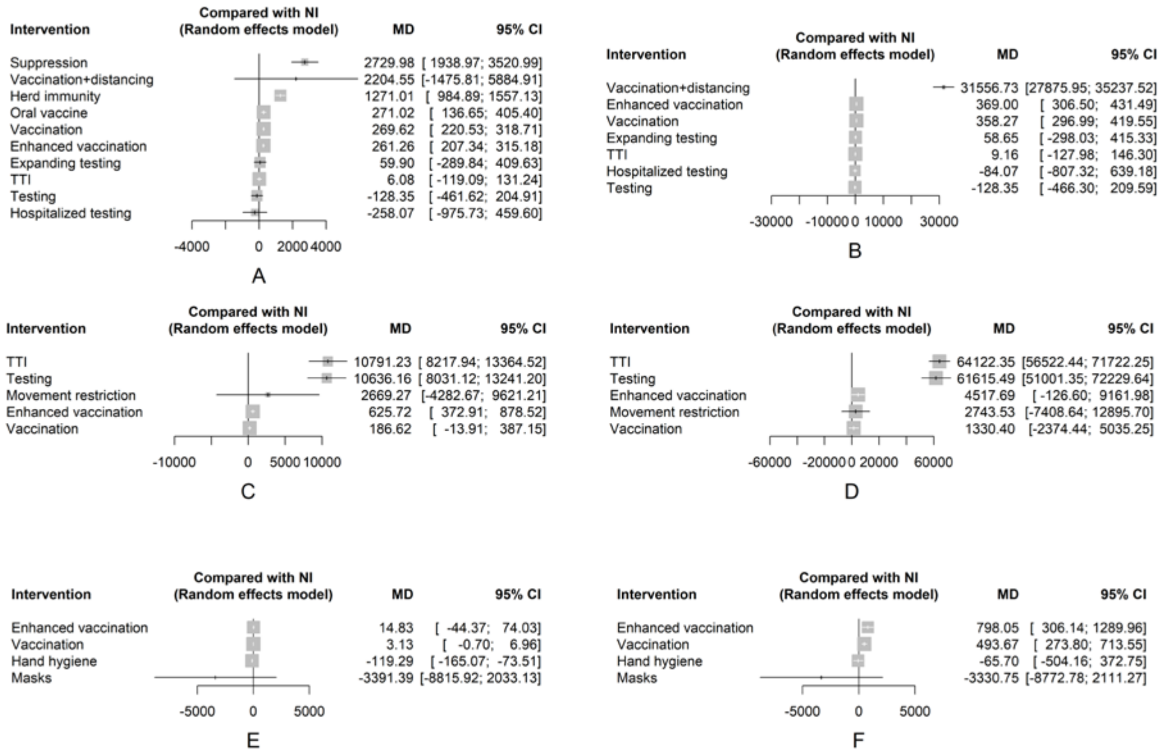

References

1. Atherly AJ, van den Broek-Altenburg EM. The effect of medical innovation on the cost-effectiveness of Covid 19-related policies in the United States using a SIR model. *BMC Health Serv Res.* 2023;23(1):372.
2. Augustovski F, Bardach A, Santoro A, et al. Cost-effectiveness of COVID-19 vaccination in Latin America and the Caribbean: an analysis in Argentina, Brazil, Chile, Colombia, Costa Rica, Mexico, and Peru. *Cost Eff Resour Alloc.* 2023;21(1):21.
3. Bagepally BS, Haridoss M, Natarajan M, Jeyashree K, Ponnaiah M. Cost-effectiveness of surgical mask, N-95 respirator, hand-hygiene and surgical mask with hand hygiene in the prevention of COVID-19: Cost effectiveness analysis from Indian context. *Clin Epidemiol Glob Health.* 2021;10:100702.
4. Bartsch SM, Weatherwax C, Martinez MF, et al. Cost-effectiveness of severe acute respiratory coronavirus virus 2 (SARS-CoV-2) testing and isolation strategies in nursing homes. *Infect Control Hosp Epidemiol.* 2024:1-8.
5. Broughel J, Kotrous M. The benefits of coronavirus suppression: A cost-benefit analysis of the response to the first wave of COVID-19 in the United States. *PLoS One.* 2021;16(6):e0252729.
6. Cai CGX, Lim NW, Huynh VA, et al. Economic Analysis of Border Control Policies during COVID-19 Pandemic: A Modelling Study to Inform Cross-Border Travel Policy between Singapore and Thailand. *Int J Environ Res Public Health.* 2023;20(5).
7. Choi W, Shim E. Assessing the cost-effectiveness of annual COVID-19 booster vaccination in South Korea using a transmission dynamic model. *Front Public Health.* 2023;11:1280412.
8. Cook DC, Fraser RW, McKirdy SJ. A benefit-cost analysis of different response scenarios to COVID-19: A case study. *Health Sci Rep.* 2021;4(2):e286.
9. Debrabant K, Gronbaek L, Kronborg C. The Cost-Effectiveness of a COVID-19 Vaccine in a Danish Context. *Clin Drug Invest.* 2021;41(11):975-988.
10. Di Fusco M, Marczell K, Thoburn E, Wiemken TL, Yang J, Yarnoff B. Public health impact and economic value of booster vaccination with Pfizer-BioNTech COVID-19 vaccine, bivalent (Original and Omicron BA.4/BA.5) in the United States. *J Med Econ.* 2023;26(1):509-524.
11. Drakesmith M, Collins B, Jones A, Nnoaham K, Thomas DR. Cost-effectiveness of a whole-area testing pilot of asymptomatic SARS-CoV-2 infections with lateral flow devices: a modelling and economic analysis study. *BMC Health Serv Res.* 2022;22(1):1190.
12. Du Z, Wang L, Bai Y, et al. Cost effectiveness of fractional doses of COVID-19 vaccine boosters in India. *Med.* 2023;4(3):182-190.e183.
13. Du Z, Wang L, Pandey A, et al. Modeling comparative cost-effectiveness of SARS-CoV-2 vaccine dose fractionation in India. *Nat Med.* 2022;28(5):934-938.
14. Du Z, Wang L, Bai Y, et al. Cost-effective proactive testing strategies during COVID-19 mass vaccination: A modelling study. *Lancet Reg Health Am.* 2022;8:100182.
15. Du Z, Pandey A, Bai Y, et al. Comparative cost-effectiveness of SARS-CoV-2 testing strategies in the USA: a modelling study. *Lancet Public Health.* 2021;6(3):e184-e191.
16. Fernandes RRA, Santos MDS, Magliano C, et al. Cost Utility of Vaccination Against COVID-19 in Brazil. *Value Health Reg Issues.* 2022;31:18-24.
17. Ferranna M, Robinson LA, Cadarette D, Eber MR, Bloom DE. The benefits and costs of U.S. employer COVID-19 vaccine mandates. *Risk Anal.* 2023.
18. Fu Y, Zhao J, Wei X, et al. Cost-Effectiveness of COVID-19 Sequential Vaccination Strategies in Inactivated Vaccinated Individuals in China. *Vaccines (Basel).* 2022;10(10).
19. Fust K, Joshi K, Beck E, et al. The potential economic impact of the updated COVID-19 mRNA fall 2023 vaccines in Japan. *Vaccines.* 2024;12(4):434.
20. Gandjour A. Was EU's COVID-19 vaccine procurement strategy irrational? A re-analysis based on cost-effectiveness considerations. *BMC Health Serv Res.* 2022;22(1):1410.
21. Gandjour A. Cost-effectiveness of future lockdown policies against the COVID-19 pandemic. *Health Serv Manage Res.* 2023;36(1):51-62.

22. González López-Valcárcel B, Vallejo-Torres L. The costs of COVID-19 and the cost-effectiveness of testing. *Applied Economic Analysis.* 2021;29(85):77-89.
23. Guzmán Ruiz Y, Vecino-Ortiz AI, Guzman-Tordecilla N, et al. Cost-Effectiveness of the COVID-19 Test, Trace and Isolate Program in Colombia. *Lancet Reg Health Am.* 2022;6:100109.
24. Hagens A, İnkaya A, Yildirak K, et al. COVID-19 Vaccination Scenarios: A Cost-Effectiveness Analysis for Turkey. *Vaccines (Basel).* 2021;9(4).
25. Hanig L, Harper CD, Nock D. COVID-19 public transit precautions: Trade-offs between risk reduction and costs. *Transp Res Interdiscip Perspect.* 2023;18:100762.
26. Heatley D. A social cost-benefit framework for COVID-19 policy decisions. *New Zeal Econ Pap.* 2022;56(1):41-48.
27. Jiang Y, Cai D, Shi S. Economic evaluations of inactivated COVID-19 vaccines in six Western Pacific and South East Asian countries and regions: A modeling study. *Infect Dis Model.* 2022;7(1):109-121.
28. Joshi K, Scholz S, Maschio M, et al. Clinical impact and cost-effectiveness of the updated COVID-19 mRNA Autumn 2023 vaccines in Germany. *J Med Econ.* 2024;27(1):39-50.
29. Karnon J, Afzali HHA, Bonevski B. An Economic Evaluation of Government-Funded COVID-19 Testing in Australia. *Appl Health Econ Health Policy.* 2022;20(5):681-691.
30. Kirson N, Swallow E, Lu J, et al. The societal economic value of COVID-19 vaccines in the United States. *J Med Econ.* 2022;25(1):119-128.
31. Kirwin E, Rafferty E, Harback K, Round J, McCabe C. A Net Benefit Approach for the Optimal Allocation of a COVID-19 Vaccine. *Pharmacoeconomics.* 2021;39(9):1059-1073.
32. Kohli MA, Maschio M, Joshi K, et al. The potential clinical impact and cost-effectiveness of the updated COVID-19 mRNA fall 2023 vaccines in the United States. *J Med Econ.* 2023;26(1):1532-1545.
33. Kohli M, Maschio M, Becker D, Weinstein MC. The potential public health and economic value of a hypothetical COVID-19 vaccine in the United States: Use of cost-effectiveness modeling to inform vaccination prioritization. *Vaccine.* 2021;39(7):1157-1164.
34. Kohli MA, Maschio M, Mould-Quevedo JF, Ashraf M, Drummond MF, Weinstein MC. The Cost-Effectiveness of Expanding Vaccination with a Cell-Based Influenza Vaccine to Low Risk Adults Aged 50 to 64 Years in the United Kingdom. *Vaccines (Basel).* 2021;9(6).
35. Li R, Lu P, Fairley CK, et al. Cost-Effectiveness of the Second COVID-19 Booster Vaccination in the USA. *Appl Health Econ Health Policy.* 2024;22(1):85-95.
36. Li R, Liu H, Fairley CK, et al. mRNA-based COVID-19 booster vaccination is highly effective and cost-effective in Australia. *Vaccine.* 2023;41(15):2439-2446.
37. Li R, Liu H, Fairley CK, et al. Cost-effectiveness analysis of BNT162b2 COVID-19 booster vaccination in the United States. *Int J Infect Dis.* 2022;119:87-94.
38. López Seguí F, Estrada Cuxart O, Mitjà IVO, et al. A Cost-Benefit Analysis of the COVID-19 Asymptomatic Mass Testing Strategy in the North Metropolitan Area of Barcelona. *Int J Environ Res Public Health.* 2021;18(13).
39. López F, Català M, Prats C, et al. A Cost-Benefit Analysis of COVID-19 Vaccination in Catalonia. *Vaccines (Basel).* 2021;10(1).
40. Lou J, Lim NW, Cai CGX, Dickens BSL, Huynh VA, Wee HL. Cost benefit analysis of alternative testing and quarantine policies for travelers for infection control: A case study of Singapore during the COVID-19 pandemic. *Front Public Health.* 2023;11:1101986.
41. Mar J, Ibarrondo O, Estadilla CDS, et al. Cost-Effectiveness Analysis of Vaccines for COVID-19 According to Sex, Comorbidity and Socioeconomic Status: A Population Study. *Pharmacoeconomics.* 2024;42(2):219-229.
42. Marco-Franco JE, Pita-Barros P, Gonzalez-de-Julian S, Sabat I, Vivas-Consuelo D. Simplified Mathematical Modelling of Uncertainty: Cost-Effectiveness of COVID-19 Vaccines in Spain. *Mathematics-Basel.* 2021;9(5).

43. Maya S, McCorvie R, Jacobson K, Shete PB, Bardach N, Kahn JG. COVID-19 Testing Strategies for K-12 Schools in California: A Cost-Effectiveness Analysis. *Int J Environ Res Public Health.* 2022;19(15).
44. Miles DK, Stedman M, Heald AH. "Stay at Home, Protect the National Health Service, Save Lives": A cost benefit analysis of the lockdown in the United Kingdom. *Int J Clin Pract.* 2021;75(3):e13674.
45. Mol BW, Karnon J. Strict Lockdown versus Flexible Social Distance Strategy for COVID-19 Disease: a Cost-Effectiveness Analysis. *Arch Clin Biomed Res.* 2023;7(1):58-63.
46. Morales-Zamora G, Espinosa O, Puertas E, et al. Cost-Effectiveness Analysis of Strategies of COVID-19 Vaccination in Colombia: Comparison of High-Risk Prioritization and No Prioritization Strategies With the Absence of a Vaccination Plan. *Value Health Reg Issues.* 2022;31:101-110.
47. Neilan AM, Losina E, Bangs AC, et al. Clinical Impact, Costs, and Cost-effectiveness of Expanded Severe Acute Respiratory Syndrome Coronavirus 2 Testing in Massachusetts. *Clin Infect Dis.* 2021;73(9):e2908-e2917.
48. Nguyen QD, Prokopenko M. A general framework for optimising cost-effectiveness of pandemic response under partial intervention measures. *Sci Rep.* 2022;12(1):19482.
49. Nurchis MC, Lontano A, Pascucci D, et al. COVID-19 Vaccination Campaign among the Health Workers of Fondazione Policlinico Universitario Agostino Gemelli IRCCS: A Cost-Benefit Analysis. *Int J Environ Res Public Health.* 2022;19(13).
50. Orangi S, Ojal J, Brand SP, et al. Epidemiological impact and cost-effectiveness analysis of COVID-19 vaccination in Kenya. *BMJ Glob Health.* 2022;7(8).
51. Orlewska K, Wierzba W, Śliwczynski A. Cost-effectiveness analysis of COVID-19 vaccination in Poland. *Arch Med Sci.* 2022;18(4):1021-1030.
52. Padula WV, Malaviya S, Reid NM, et al. Economic value of vaccines to address the COVID-19 pandemic: a U.S. cost-effectiveness and budget impact analysis. *J Med Econ.* 2021;24(1):1060-1069.
53. Patenaude B, Ballreich J. Modeling the potential economic benefits of an oral SARS-CoV-2 vaccine during an outbreak of COVID-19. *BMC Public Health.* 2022;22(1):1792.
54. Pearson CAB, Bozzani F, Procter SR, et al. COVID-19 vaccination in Sindh Province, Pakistan: A modelling study of health impact and cost-effectiveness. *PLoS Med.* 2021;18(10):e1003815.
55. Reddy KP, Fitzmaurice KP, Scott JA, et al. Clinical outcomes and cost-effectiveness of COVID-19 vaccination in South Africa. *Nat Commun.* 2021;12(1):6238.
56. Reddy KP, Shebl FM, Foote JHA, et al. Cost-effectiveness of public health strategies for COVID-19 epidemic control in South Africa: a microsimulation modelling study. *Lancet Glob Health.* 2021;9(2):e120-e129.
57. Ruiz FJ, Torres-Rueda S, Pearson CAB, et al. What, how and who: Cost-effectiveness analyses of COVID-19 vaccination to inform key policies in Nigeria. *PLOS Glob Public Health.* 2023;3(3):e0001693.
58. Sandmann FG, Davies NG, Vassall A, Edmunds WJ, Jit M, Centre for the Mathematical Modelling of Infectious Diseases C-wg. The potential health and economic value of SARS-CoV-2 vaccination alongside physical distancing in the UK: a transmission model-based future scenario analysis and economic evaluation. *Lancet Infect Dis.* 2021;21(7):962-974.
59. Scherbina A. Assessing the Optimality of a COVID Lockdown in the United States. *Econ Disaster Clim Chang.* 2021;5(2):177-201.
60. Schonberger RB, Listokin YJ, Ayres I, Yaesoubi R, Shelley ZR. Cost Benefit Analysis of Limited Reopening Relative to a Herd Immunity Strategy or Shelter in Place for SARS-CoV-2 in the United States. *medRxiv.* 2020.
61. Sirison K, Nittayasoot N, Techasuwanna R, Cetthakrikul N, Suphanchaimat R. Cost-Effectiveness Analysis of COVID-19 Vaccine Booster Dose in the Thai Setting during the Period of Omicron Variant Predominance. *Trop Med Infect Dis.* 2023;8(2).

62. Suwantika AA, Dhamanti I, Suharto Y, Purba FD, Abdulah R. The cost-effectiveness of social distancing measures for mitigating the COVID-19 pandemic in a highly-populated country: A case study in Indonesia. *Travel Med Infect Dis.* 2022;45:102245.
63. Szanyi J, Wilson T, Howe S, et al. Epidemiologic and economic modelling of optimal COVID-19 policy: public health and social measures, masks and vaccines in Victoria, Australia. *Lancet Reg Health West Pac.* 2023;32:100675.
64. Tan C, Luo X, Zhou Z, et al. Dynamic zero-COVID strategy in controlling COVID-19 in Shanghai, China: A cost-effectiveness analysis. *J Infect Public Health.* 2023;16(6):893-900.
65. Tuite AR, Ng V, Ximenes R, et al. Quantifying the economic gains associated with COVID-19 vaccination in the Canadian population: A cost-benefit analysis. *Can Commun Dis Rep.* 2023;49(6):263-273.
66. Vaezi A, Meysamie A. COVID-19 Vaccines Cost-Effectiveness Analysis: A Scenario for Iran. *Vaccines (Basel).* 2021;10(1).
67. Vilches TN, Rafferty E, Wells CR, Galvani AP, Moghadas SM. Economic evaluation of COVID-19 rapid antigen screening programs in the workplace. *BMC Med.* 2022;20(1):452.
68. Wang WC, Fann JC, Chang RE, et al. Economic evaluation for mass vaccination against COVID-19. *J Formos Med Assoc.* 2021;120 Suppl 1:S95-s105.
69. Wang X, Cai Y, Zhang B, et al. Cost-effectiveness analysis on COVID-19 surveillance strategy of large-scale sports competition. *Infect Dis Poverty.* 2022;11(1):32.
70. Wang Y, Luangasanatip N, Pan-Ngum W, et al. Assessing the cost-effectiveness of COVID-19 vaccines in a low incidence and low mortality setting: the case of Thailand at start of the pandemic. *Eur J Health Econ.* 2022:1-14.
71. Xiong X, Li J, Huang B, et al. Economic Value of Vaccines to Address the COVID-19 Pandemic in Hong Kong: A Cost-Effectiveness Analysis. *Vaccines (Basel).* 2022;10(4).
72. Zafari Z, de Oliveira PM, Gkantonas S, Ezeh C, Muennig PA. The cost-effectiveness of standalone HEPA filtration units for the prevention of airborne SARS CoV-2 transmission. *Cost Eff Resour Alloc.* 2022;20(1):22.
73. Zafari Z, Goldman L, Kovrizhkin K, Muennig PA. The cost-effectiveness of common strategies for the prevention of transmission of SARS-CoV-2 in universities. *PLoS One.* 2021;16(9):e0257806.
74. Zhao J, Jin H, Li X, et al. Disease Burden Attributable to the First Wave of COVID-19 in China and the Effect of Timing on the Cost-Effectiveness of Movement Restriction Policies. *Value Health.* 2021;24(5):615-624.
75. Zhou H, Ding N, Han X, et al. Cost-effectiveness analysis of vaccination against COVID-19 in China. *Front Public Health.* 2023;11:1037556.
76. Xiao H, Liu F, Unger JM. Dynamic zero-COVID policy and healthcare utilization patterns in China during the Shanghai COVID-19 Omicron outbreak. *Commun Med (Lond).* 2023;3(1):143.
